# Polymicrobial Aggregates in Human Saliva Build the Oral Biofilm

**DOI:** 10.1128/mbio.00131-22

**Published:** 2022-02-22

**Authors:** Aurea Simon-Soro, Zhi Ren, Bastiaan P. Krom, Michel A. Hoogenkamp, Pedro J. Cabello-Yeves, Scott G. Daniel, Kyle Bittinger, Inmaculada Tomas, Hyun Koo, Alex Mira

**Affiliations:** a Biofilm Research Laboratories, Department of Orthodontics, Divisions of Community Oral Health & Pediatric Dentistry, School of Dental Medicine, University of Pennsylvania, Philadelphia, Pennsylvania, USA; b Center for Advanced Research in Public Health, FISABIO Foundation, Valencia, Spain; c Department of Preventive Dentistry, Academic Centre for Dentistry Amsterdam, University of Amsterdam and Vrije Universiteit Amsterdam, Amsterdam, The Netherlands; d Division of Gastroenterology, Hepatology, and Nutrition, Children's Hospital of Philadelphia, Philadelphia, Pennsylvania, USA; e Oral Sciences Research Group, Special Needs Unit, Department of Surgery and Medical-Surgical Specialties, School of Medicine and Dentistry, Universidade de Santiago de Compostela, Health Research Institute Foundation of Santiago (FIDIS), Santiago de Compostela, Spain; f Center for Innovation & Precision Dentistry, School of Dental Medicine and School of Engineering & Applied Sciences, University of Pennsylvania, Philadelphia, Pennsylvania, USA; g CIBER Center for Epidemiology and Public Health, Madrid, Spain; Georgia Institute of Technology School of Biological Sciences

**Keywords:** polymicrobial aggregate, saliva, oral biofilm, spatial structure, microbiome

## Abstract

Biofilm community development has been established as a sequential process starting from the attachment of single cells on a surface. However, microorganisms are often found as aggregates in the environment and in biological fluids. Here, we conduct a comprehensive analysis of the native structure and composition of aggregated microbial assemblages in human saliva and investigate their spatiotemporal attachment and biofilm community development. Using multiscale imaging, cell sorting, and computational approaches combined with sequencing analysis, a diverse mixture of aggregates varying in size, structure, and microbial composition, including bacteria associated with host epithelial cells, can be found in saliva in addition to a few single-cell forms. Phylogenetic analysis reveals a mixture of complex consortia of aerobes and anaerobes in which bacteria traditionally considered early and late colonizers are found mixed together. When individually tracked during colonization and biofilm initiation, aggregates rapidly proliferate and expand tridimensionally, modulating population growth, spatial organization, and community scaffolding. In contrast, most single cells remain static or are incorporated by actively growing aggregates. These results suggest an alternative biofilm development process whereby aggregates containing different species or associated with human cells collectively adhere to the surface as “growth nuclei” to build the biofilm and shape polymicrobial communities at various spatial and taxonomic scales.

## INTRODUCTION

Biofilms are highly organized microbial communities residing on surfaces (1–4). With few exceptions, biofilms harbor polymicrobial communities displaying complex spatial organization and interspecies interactions. Extensive efforts have been devoted to understanding the biofilm initiation and further development (5, 6). A key step is the initial attachment of microbial cells on a surface. Free-living bacteria (planktonic cells) can bind to the surface and start the formation of a matrix-enclosed microbial community, creating its own microenvironment, which varies depending on the host or other natural niches. The common model relies on a single species or a community of different species binding to the surface as single cells (7). However, microorganisms in natural settings or in biological fluids are often found forming multicellular aggregates in addition to planktonic cells (8–10). Bacteria in saliva or in synovial fluid usually form aggregates (11–13), while aggregated microbes are also found in aquatic ecosystems (14). Recent studies have assessed the role of aggregates in biofilm formation using individual single-species laboratory strains (8, 15). However, how naturally occurring aggregates modulate biofilm initiation and community development remains largely unexplored.

Human saliva harbors a myriad of microbial species that interact and bind to several surfaces in the oral cavity, leading to the initiation of biofilms. Oral biofilms are one of the most diverse communities that have provided fundamental knowledge about biofilm biology and development (6). Microbes can contribute to oral and systemic health, but they also mediate oral diseases such as dental caries and periodontitis. Daily oral hygiene measures help to remove oral biofilms from the tooth surface. However, oral biofilms rapidly return, and the ecological succession model, whereby individual species colonize tooth surfaces in a sequential and orderly manner, has been widely demonstrated and adopted (1, 16–24). In this model, microbes are grouped into early colonizers (those that bind to the tooth surface within the first hours), which can provide attachment sites for other species, and late colonizers, which increase after days or weeks of biofilm development (25). Recent advances combining micron-scale biogeography and high-throughput sequencing enhanced the understanding of the spatial and phylogenetic scales of biofilm community arrangement (1, 26, 27).

Analysis of dental biofilms (plaque) in their native state from clinical samples revealed a more complex structure and composition than previously considered, harboring spatially heterogeneous clusters where bacteria traditionally considered early colonizers occupy external positions in the biofilm (1, 3, 4), which is difficult to explain under the current model. Likewise, a diverse mixture of early and late colonizers can be found in biofilms *in vivo* (21, 22), suggesting a less ordered pattern of the initial colonizing bacteria. Although microbial aggregates were found in saliva decades ago, their structure, taxonomic organization, and roles in biofilm initiation and structuring have not been explored. Further elucidation on whether native-state aggregates in human saliva can influence biofilm initiation at spatiotemporal and phylogenetic scales may provide new fundamental insights.

Here, we comprehensively analyze the composition and structure of the human-derived aggregated salivary community in its native state and their role in biofilm initiation using multiscale time-lapsed imaging, flow cytometry, and sequencing with computational analyses across different time points. We find naturally occurring aggregates with various sizes, structures, and compositions, including cobinding with human cells and free-living cells. In addition, early and late colonizers are found together in mixed-species aggregates. By tracking the entire population of surface colonizers, we discover that polymicrobial aggregates actively bind and rapidly proliferate, whereas single bacterial cells remain mostly static. Further analyses reveal that the aggregated consortia act as growth nuclei expanding across the surface, engulfing the static single cells to coordinate the composition, size, and diversity of the biofilm community structure. Hence, our results suggest an addition to classical models of sequential single-cell binding and orderly ecological succession, whereby mixed microbes collectively adhere as buds of growth, leading to spatially and taxonomically heterogeneous communities, which may be applicable to other microbiomes harboring similar polymicrobial aggregates.

## RESULTS

### Native-state microbial structure in human saliva.

**FIG 1:**
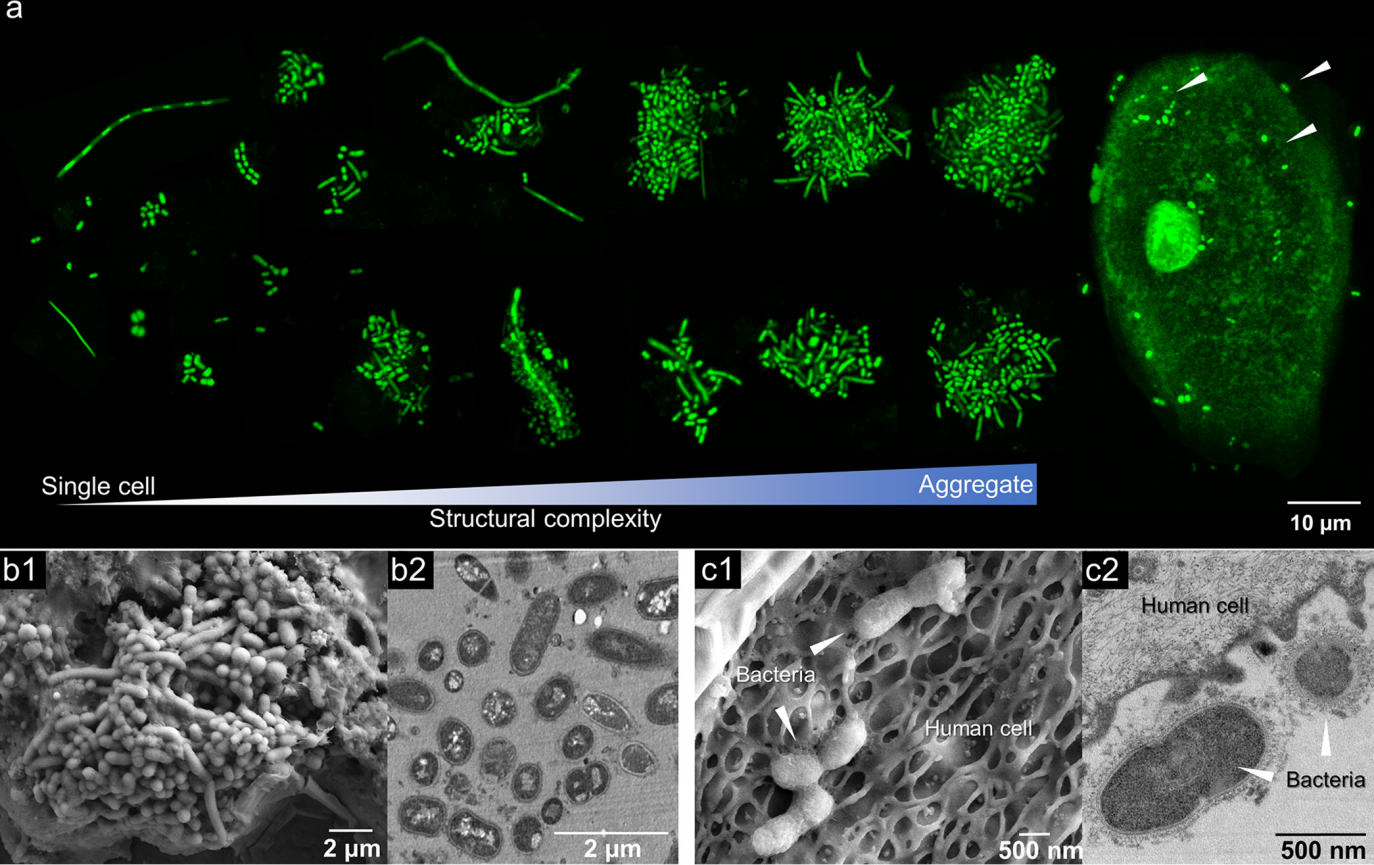
Microbial community structure in human saliva in its native state. (a) Confocal microscopy of the structured microorganisms found in intact human saliva showing a highly complex mixture of single cells, microbial aggregates, and bacteria attached to host epithelial cells (arrowheads). (b) Scanning electron microscopy (b1) and transmission electron microscopy (b2) of polymicrobial aggregates found in human saliva. (c) Microorganisms in human saliva associated with desquamated oral epithelial cells as shown by SEM (c1) and TEM (c2) images.

We performed a comprehensive analysis of the structure and composition of the salivary microbial community in its native, intact form using a combination of super-resolution confocal imaging and scanning/transmission electron microscopy (SEM and TEM). We found that the microorganisms in human saliva rarely appeared in a single-cell free-living state. Rather, the microbes formed clusters, including some associated with desquamated oral epithelial cells (Fig. 1). The microbial aggregates in saliva ranged from 3 to 10 μm up to 50 μm in diameter.

**FIG 2:**
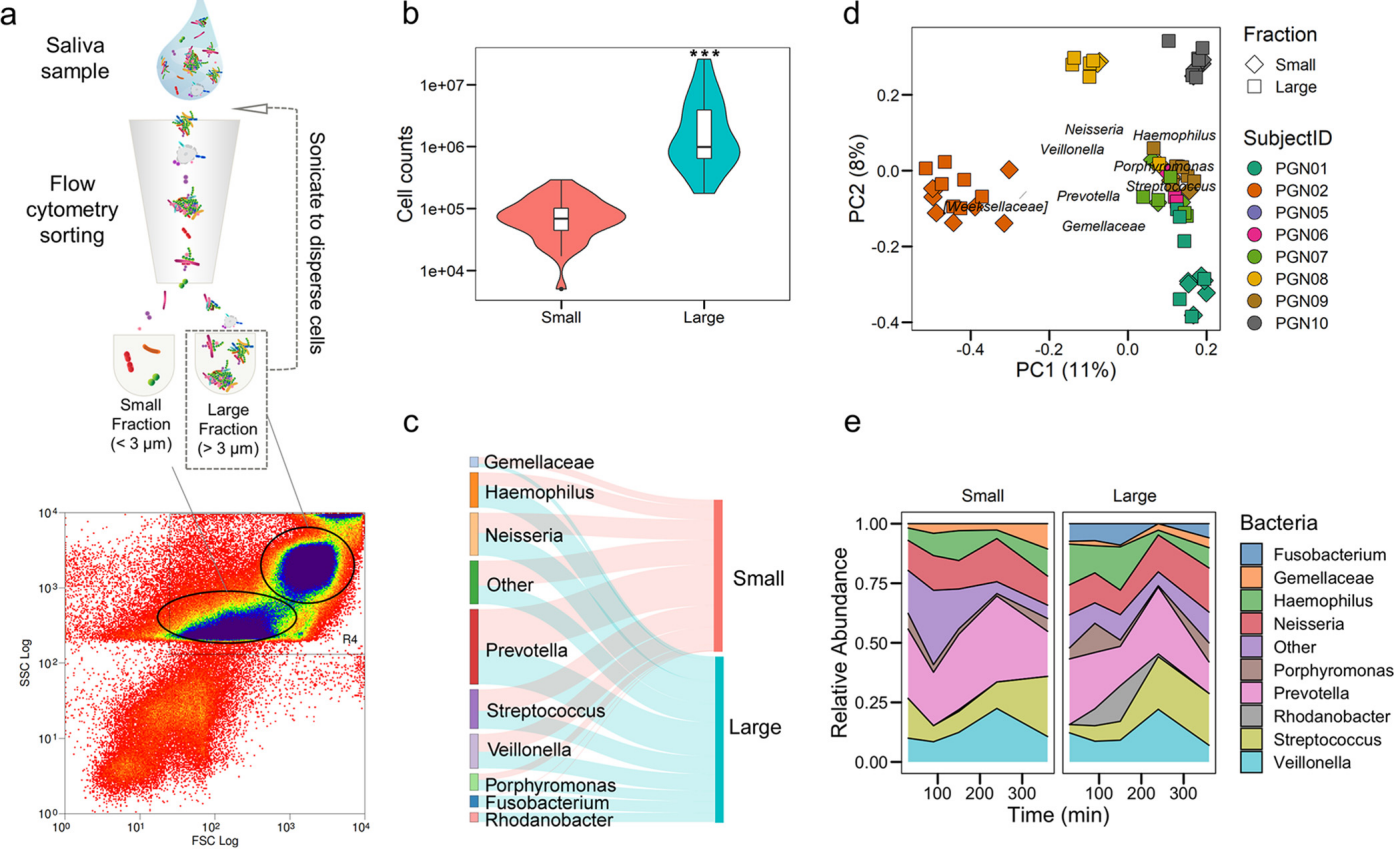
Bacterial composition within community structure in saliva. (a) Diagram of the workflow to assess and sort the microbial structures in saliva using flow cytometry. We sorted the sample into two subgroups based on the clustering of the flow cytometry data, described as small (<3 μm) and large (>3 μm) fractions. For cell counting, the large fraction was sonicated to disperse microbial aggregates and reanalyzed by flow cytometry. (b) Cell counts of the sorted cells (event numbers) in small and large fractions of saliva samples. (c) Sankey diagram showing bacterial composition abundance (line thickness) in each sorted fraction. (d) Principal correspondence analysis using Jaccard index analysis of the small and large (shapes) fractions of 8 subjects (color). (e) Bacterial abundance dynamics on each sorted fraction found through time of sample collection (0 to 360 min). ***, *P < *0.001 by Wilcoxon test.

We then used flow cytometry to investigate the size and complexity of the microbial structures in saliva from human subjects (20). Saliva samples were collected 30, 90, 150, and 360 min after toothbrushing. Fluorescence-activated cell sorting was employed to separate the different microbial constituents by sorting based on complexity (Fig. 2a). Data analysis revealed that, based on size, microbial structures in saliva clustered into 2 subpopulations, corresponding to a small fraction (<3 μm) and a large fraction (>3 μm). Microscopic examination indicated that the fraction under 3 μm contained mostly single cells. In contrast, we observed a highly heterogeneous population within the large fraction (>3 μm), including aggregates composed of different bacterial cells and desquamated epithelial cells with bound microorganisms. The proportion of free-living cells was 3%, whereas 97% of microorganisms were found as aggregates based on cell counts in each fraction (see Table S1 in the supplemental material), indicating that the microorganisms in saliva were predominantly in aggregated form (versus planktonic single cells; *P* *< *0.001) (Fig. 2b).

10.1128/mbio.00131-22.7 TABLE S1 Cell event counts in small/large fractions of saliva from human subjects. Data from samples collected at different time points (30 min, 90 min, 150 min, and 360 min after toothbrushing) are included. Data are presented as mean, minimum (Min), maximum (Max), standard deviations (SD), and percentage. Download Table S1, PDF file, 0.1 MB. Copyright © 2022 Simon-Soro and Ren et al. 2022 Simon-Soro and Ren et al. https://creativecommons.org/licenses/by/4.0/ This content is distributed under the terms of the Creative Commons Attribution 4.0 International license.

### Bacterial composition within community structure in saliva.

Next, we asked whether the salivary microbial compositions in single-cell and aggregated forms are different. Using 16S rRNA sequencing, we analyzed the bacterial composition in the small and large fractions sorted by flow cytometry. We observed that specific taxa can be found in aggregates such as *Porphyromonas*, *Fusobacterium*, and Haemophilus, whereas other species can be found as both free-living single cells and in aggregated communities, including *Prevotella*, *Neisseria*, Streptococcus, and *Veillonella* (Fig. 2c). We then asked whether the different microbial species composition in the two sorted fractions was driven by the composition and distribution of the bacteria across all the subjects. We analyzed the beta-diversity using Jaccard distances and observed that samples clearly clustered by subject but not by community structure (Fig. 2d), indicating that both small and large fractions had similar bacterial compositions. Considering the salivary circadian rhythm might affect the structure and composition of the microorganisms, we also assessed both the small and large fractions at different time points. Our data suggested that the bacterial composition was variable between individuals and through time (Fig. 2e). Virtually all genera appeared to be present already at 30 min after toothbrushing (morning), including strict anaerobes traditionally considered late colonizers, like *Prevotella* or *Veillonella*. Genera classically considered early colonizers, like Streptococcus, *Granulicatella*, and *Gemella*, were found at higher levels after 6 h (afternoon). Interestingly, we observed some individuals showed a very constant pattern of bacterial composition in the different salivary size fractions and through time (PGN08), whereas others showed several bacterial genera preferentially found in aggregates or in the planktonic fraction (PGN09), with some temporal changes (Fig. S3a).

10.1128/mbio.00131-22.3 FIG S3 (a) Subject specific microbial abundances by saliva fraction. Alluvial plot visualizes the bacterial abundance (flow line thickness) found on small and large fractions in sorted saliva samples for each subject. Samples were taking at 30, 90, 150, and 360 min after toothbrushing. (b) Active bacteria in supragingival dental plaque (360 min after toothbrushing). Bacterial composition was determined by high-throughput sequencing of the 16S rRNA gene after RNA extraction. Heatmap shows the proportion of the main active bacteria in the dental plaque metatranscriptome at 360 min posttoothbrushing on 14 subjects. Download FIG S3, PDF file, 0.4 MB. Copyright © 2022 Simon-Soro and Ren et al. 2022 Simon-Soro and Ren et al. https://creativecommons.org/licenses/by/4.0/ This content is distributed under the terms of the Creative Commons Attribution 4.0 International license.

Despite individual variability, the overall microbial distribution and composition were conserved, showing that in saliva (i) microbes are present primarily in the large (aggregate) fraction rather than in the small (single-cell) fraction, (ii) certain bacteria form primarily aggregates while others can be in both single-cell state and aggregated forms, and (iii) bacteria that traditionally have been considered late and early colonizers can be found mixed in the aggregate fraction. In addition, a preliminary analysis of the microbial composition of initially formed dental plaque samples (6 h posttoothbrushing) show that both early and late colonizers are all detected together (Fig. S3b), suggesting that they may bind concomitantly as mixed consortia to modulate biofilm formation. Hence, we explored whether single cells or aggregates preferentially attach to a hydroxyapatite surface to initiate and develop the biofilm.

### Seeding of saliva-derived biofilms.

Biofilm formation involves bacterial colonization and further growth of the colonizers on a surface (6). The presence of aggregated microbial communities in saliva indicates that these structures are members of the initial colonizing community. To investigate whether aggregates in their native state form biofilms, we developed an *ex vivo* fluid-to-biofilm model using time-lapse microscopy within a microfluidics system combined with computational analyses (see Materials and Methods). We first characterized the microbial binding pattern (initial colonizers) on the hydroxyapatite surface (HA; a tooth enamel surrogate). Given that the microbial composition of saliva varies in different donors, we used pooled unstimulated saliva to assess the spatial distribution of initial microbial colonization to HA. The HA was incubated with the pooled saliva inoculum to allow bacterial binding before being aseptically transferred to a microfluidic imaging device. The HA surface was analyzed by confocal imaging of the colonized structures at various time points (30 to 240 min). By comparing the surface coverage rates, we first determined that maximum binding was reached after 60 min of incubation (Fig. S1). We then assessed the composition of microbial species in the fluid phase (saliva inoculum) and solid phase (surface bound).

10.1128/mbio.00131-22.1 FIG S1 Surface coverage by microorganisms in biofilms formed from saliva inoculum after 30 min, 60 min, 120 min, and 240 min. Surface coverage by the colonizing microbial community was determined using confocal microscopy and computational image analysis with BiofilmQ. Maximum binding with the highest coverage was reached after 60 min of incubation, which was used for further experiments. *, *P < *0.05, one-way analysis of variance with Dunnett’s multiple-comparison test. NS, not significantly different. Download FIG S1, PDF file, 0.1 MB. Copyright © 2022 Simon-Soro and Ren et al. 2022 Simon-Soro and Ren et al. https://creativecommons.org/licenses/by/4.0/ This content is distributed under the terms of the Creative Commons Attribution 4.0 International license.

**FIG 3:**
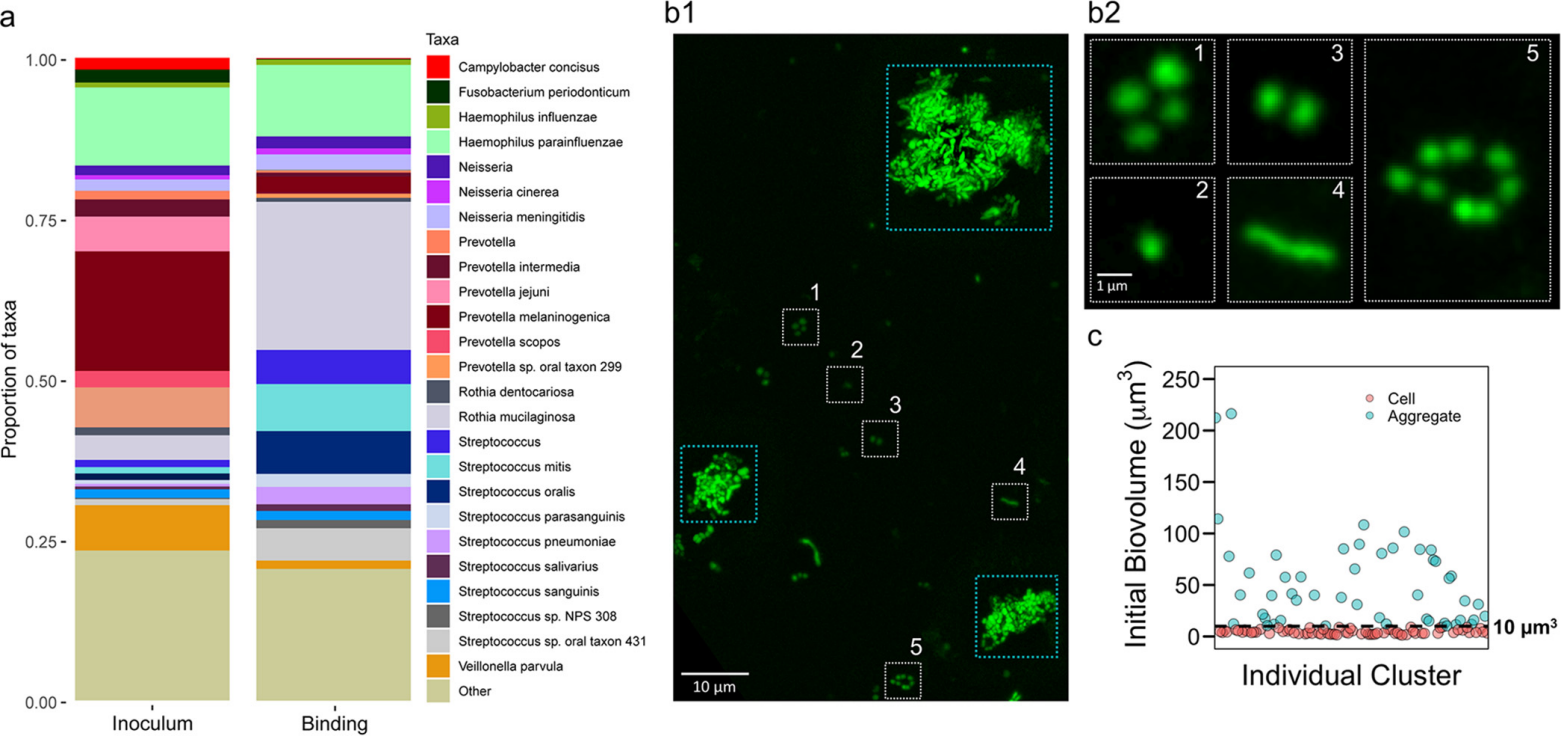
Seeding of saliva-derived biofilms. (a) Microbial species found in the fluid phase (saliva inoculum) and solid phase (binding). Low-frequency bacteria are represented as “Other” and include Treponema denticola, *Aggregatibacter actinomycetencomitans*, Tannerella forsythia, and Porphyromonas gingivalis. Early colonizers (e.g., Streptococcus, Haemophilus, *Capnocythophaga*, *Actinomyces*, and *Veillonella*) together with species traditionally considered late colonizers (e.g., *Prevotella*, *Porphyromonas*, and Treponema) were found on the surface after the initial binding, suggesting mixed-species cocolonization during the binding process. Fusobacterium nucleatum, a bridging organism, was also found in this initial colonizing community. Data represent pooled samples from three individuals, and three independent experiments were performed. (b) Representative image (Z projection) of *in situ* visualization of surface colonizing units on the saliva-coated tooth-mimetic hydroxyapatite surface. (b1) A highly diverse and heterogenous structural organization, including both single cells/clusters (in white box) and large aggregates (in cyan box), can bind to the surface. (b2) Magnified images of the single cells/small clusters (as shown in panel b1). (c) Initial biovolume of the individual colonizing units was analyzed using BiofilmQ software based on the 3-dimensional confocal data sets. A threshold biovolume (*V*) of 10 μm^3^ (black dotted line) was used to group the initial colonizing units: small clusters with *V* of * *≤10 μm^3^ were defined as single cells (depicted in pink), and large clusters with *V* of >10 μm^3^ were defined as aggregates (blue). The results represent a large population of initial colonizing units (*N* > 300). Imaging and metagenomics data were obtained from at least 3 independent experiments.

Notably, we found that a diverse mixture of traditionally described early and late colonizers (17–20, 26) in saliva could bind simultaneously on the HA surface (Fig. 3a and Fig. S4). Early colonizers detected in our model include Streptococcus, Haemophilus, *Capnocytophaga*, *Actinomyces*, and *Veillonella*, whereas late colonizers, such as *Prevotella*, Treponema, *Aggregatibacter*, and *Porphyromonas*, were also attached on the apatitic surface, suggesting mixed-species cocolonization during the binding process. Fusobacterium nucleatum, a previously described bridging organism, was also found in this initial colonizing community. The entire population of surface colonizers was then imaged using our optimized biofilm imaging platform (4, 26) at multiple length scales. We found a large population of surface-bound bacteria (*n* > 50 in an area of 319.45 by 319.45 μm^2^), comprised of a diverse mixture of single cells, bacterial chains, and highly heterogeneous microbial aggregates, as initial colonizing units (Fig. 3b1 and b2). We employed BiofilmQ (28) to characterize the individual structures of the colonizing community. This was performed by segmenting the image subsets containing individual units at the regions where the bacteria attached after the 60 min binding period (*t*_0_). The biovolumes of individual units varied from ∼2 to 250 μm^3^ (Fig. 3c). We further grouped all the initial colonizing units based on their biovolume (*V*): small clusters with *V* of ≤10 μm^3^ were defined as single cells (26), and large clusters with *V* of >10 μm^3^ were defined as aggregates (Fig. 3c). The data show a remarkably diverse colonizing community (or colonizing units) comprised not only of single cells but also aggregates of various sizes and shapes bound on the apatitic surface. The initial colonizing units may provide distinctive starting points for further biofilm growth.

10.1128/mbio.00131-22.4 FIG S4 Microbial species in the saliva inoculum and at binding. The abundance of each species on the *y* axis is relative to the total amount of either early (top) or late colonizers (bottom). Early colonizers detected include Streptococcus, Haemophilus, *Capnocythophaga*, *Actinomyces*, and *Veillonella*, whereas species that are traditionally considered late colonizers, such as *Prevotella*, Treponema, *Aggregatibacter*, and *Porphyromonas*, were also found in the initial colonizing community, suggesting mixed-species cocolonization during the binding process. Fusobacterium nucleatum, a bridging organism, was also found in this initial colonizing community. Download FIG S4, PDF file, 0.2 MB. Copyright © 2022 Simon-Soro and Ren et al. 2022 Simon-Soro and Ren et al. https://creativecommons.org/licenses/by/4.0/ This content is distributed under the terms of the Creative Commons Attribution 4.0 International license.

### Dynamics of biofilm initiation and growth from saliva fluid.

**FIG 4:**
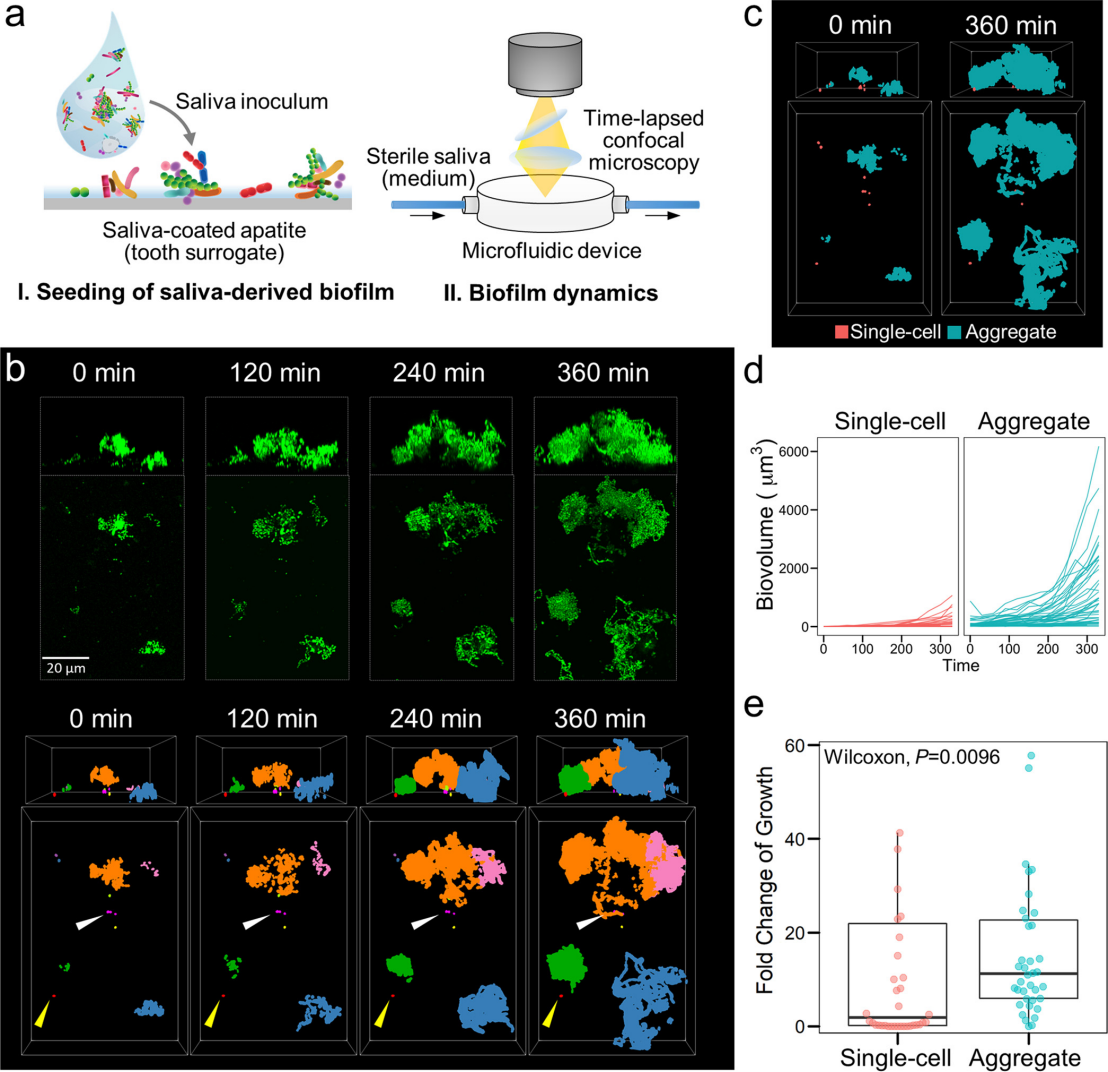
Dynamics of biofilm initiation and growth from saliva fluid. (a) Experimental setup for fluid-to-biofilm model used for assessing the colonization of microorganisms in saliva fluid (*t* = 0 min) and further growth under cell-free saliva flow in real time. (b, upper) Time-lapsed confocal images (represented as Z projection and orthogonal projection) of biofilm initiation from saliva fluid. (Lower) Three-dimensional time-lapsed tracking map of biofilm growth. Colors indicate individual biofilm clusters that originated from different initial colonizing units. Only the clusters that attached at the initial binding (*t* = 0 min) and remained bound until the end of the experiment (*t* = 360 min) were tracked. Yellow arrow, a colonized single cell that stayed static; white arrow, attached single cells that remained static and were engulfed by a growing aggregate. (c) The spatial distribution of biofilm growth by origin (single cell or aggregate). Blue, biovolume originated from aggregates; pink, biovolume originated from single cells. (d) Growth curves of individual colonizing units within 360 min after initial colonization. (Left) Most single cells did not grow or grew slowly despite remaining attached to the surface (depicted in pink). (Right) Most initial bound aggregates grew steadily, with some reaching large sizes (depicted in blue). (e) Fold change of single cell or aggregate growth.

Considering that both aggregates and single cells are present in saliva and bound to apatitic surfaces, we investigated how this colonizing community mediates biofilm initiation spatiotemporally. To investigate the growth dynamics of surface-attached saliva-derived microbes, we applied a high-resolution confocal imaging system coupled with flow-cell microfluidics that was optimized for biofilm time-lapsed imaging (Fig. 4a) (26). We used filter-sterilized pooled saliva as the culture medium for the fluid-to-biofilm model to mimic the natural nutrient condition in the oral cavity. Upon initiating the flow in the microfluidic device, biofilm growth was observed across the HA, and image acquisition was performed every 30 min to generate a 4-dimensional data set (*x*, *y*, *z*, and time frame) for further analysis (Fig. 4b, top, and Movie S1). We found that a subset of the initial colonizing units actively grew and evolved into larger biofilm structures (Movie S1).

10.1128/mbio.00131-22.8 MOVIE S1 Dynamics of biofilm initiation from saliva fluid in the fluid-to-biofilm model. Left, time-lapsed confocal images (represented as Z projection and orthogonal projection) of biofilm initiation from saliva fluid. Right, three-dimensional time-lapsed tracking map of the biofilm growth. Colors indicate individual biofilm clusters that originated from different initial colonizing units. Only the clusters that attached at the initial binding (*t *= 0 min) and remained bound until the end of the experiment (*t* = 360 min) were tracked. A yellow arrow indicates a colonized single cell that stayed static. A white arrow indicates attached single cells that remained static and were engulfed by a growing aggregate. Scale bar, 20 μm. Download Movie S1, MOV file, 1.1 MB. Copyright © 2022 Simon-Soro and Ren et al. 2022 Simon-Soro and Ren et al. https://creativecommons.org/licenses/by/4.0/ This content is distributed under the terms of the Creative Commons Attribution 4.0 International license.

To investigate whether the initial structure had an impact on the growth dynamics, we applied geometrical filtering to assign all the initial colonizing units into two subgroups based on their initial biovolume (Vt_0_) as single cells (Vt_0_ ≤ 10 μm^3^) or aggregates (Vt_0_ > 10 μm^3^). Because bacterial cells can detach under flow and cause discrepancy in the calculation of the growth dynamics, we only analyzed the individual colonizing units that were attached at the initial binding and remained bound on the surface throughout the experiment (until 360 min). Each colonizing unit was monitored individually using the cube tracking algorithm in BiofilmQ. Biofilm structures originating from the same starting point (i.e., the same colonizing unit) were assigned the same track identifier (ID; color coded) so that each unit could be traced individually from the initial binding (*t*_0min_) (Fig. 4b, bottom, and Movie S1). We found that most aggregates steadily grow, whereas most single-cell units stay static (yellow arrow in Fig. 4b) or are engulfed by a growing aggregate (white arrow). To further visualize the spatial growth distribution of the colonizing units, we separated those growing from either single cells (depicted in pink) or aggregates (in blue), as shown in Fig. 4c. The data suggest that the biofilm community growth on the surface originated mostly from aggregates rather than from single cells. In addition, we also compared the total biovolume (biomass) of all tracked colonizing units from the beginning (*t*_0min_) to the end of the experiment (*t*_360min_) on an area of 159.73 by 159.73 μm^2^. As shown in Fig. S5, the total biovolume from an aggregate origin increased over time, accounting for >90% of the total amount at the endpoint (∼17,000 μm^3^). In contrast, single cell-derived biovolume remained low, resulting in less than 1,500 μm^3^ at 360 min.

10.1128/mbio.00131-22.5 FIG S5 Total biovolume of single cell/aggregate-derived biofilm. Time-resolved biofilm volume originated from colonizing units in different subgroups (single cells and aggregates) on a surface area of 159.73 by 159.73 μm^2^. Saliva-derived biofilm growth was monitored by time-lapsed confocal imaging in the fluid-to-biofilm system. Data were computationally analyzed using BiofilmQ. The total biovolume from an aggregate origin (in blue) increased over time, accounting for >90% of the total amount at the endpoint (∼17,000 μm^3^). In contrast, single-cell-derived biovolume (in pink) remained low, resulting in less than 1,500 μm^3^ at 360 min. Download FIG S5, PDF file, 0.1 MB. Copyright © 2022 Simon-Soro and Ren et al. 2022 Simon-Soro and Ren et al. https://creativecommons.org/licenses/by/4.0/ This content is distributed under the terms of the Creative Commons Attribution 4.0 International license.

Next, we further analyzed the biovolume-based growth curves of each colonizing unit and compared its dynamic changes. Our data revealed that most initial colonizing units grouped as single cells did not grow or grew slowly despite remaining attached to the surface throughout the experiment period (Fig. 4d, left, and e). Only 42% of single cells increased in biovolume with a median fold change of 1.83-fold (Fig. 4e). In contrast, most initial colonizing units bound as aggregates grew steadily (79% of aggregates increased with a median fold change of 11.2-fold; *P = *0.0096), reaching large sizes ranging between 2,000 and 6,000 μm^3^ at 360 min (Fig. 4d, right; Fig. 4e). Thus, two distinctive phenotypes were observed: (i) static or slow-growing colonizing units, predominantly single cells, whereby the bacteria bound to the HA maintained their biovolume relatively constant or with limited changes, and (ii) active-growing units, predominantly comprised of aggregates of various sizes that grew faster and expanded their biovolume over time, often engulfing the static population.

**FIG 5:**
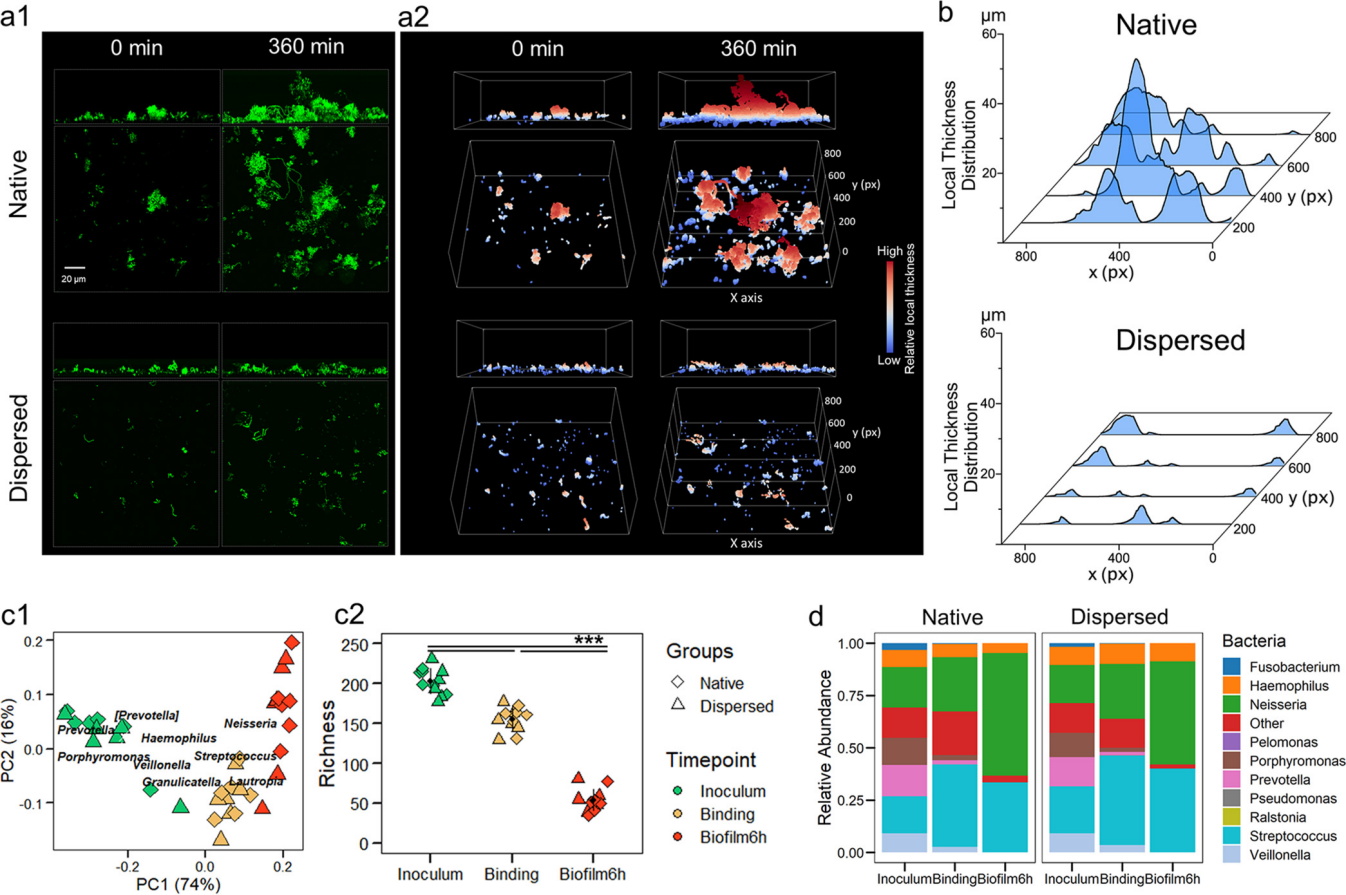
Spatial and taxonomic scale of native and dispersed communities. (a) Biofilm initiation from the native-state saliva inoculum versus the dispersed saliva inoculum. (a1) Confocal images (represented as Z projection and orthogonal projection) of biofilm originating from the native state (top) and the dispersed inoculum (bottom). (a2) Three-dimensional rendering of the *in situ* biofilm morphological analysis showing the 0-min and 360-min biofilm structures. (b) Results of biofilm thickness mapping showing the local thickness distribution at 360 min at four different cross sections (as shown in panel a2). (c1) Principal correspondence analysis using weighted UniFrac distances of the native and dispersed groups of human saliva used as inoculum and the biofilm initiation stages (binding and biofilm at 6 h). (c2) Richness analysis of the biofilm stages. Median is indicated in each group with the black symbol. *P < *0.001 by pairwise comparisons using Kruskal-Wallis H test. (d) Bacterial relative abundances of the main genera of native and dispersed groups.

Given the contrasting growth behavior between aggregated consortia and single cells, we hypothesized that the aggregated form could act as biofilm initiators promoting the spatial growth and structural heterogeneity of the microbial community. Using the same model for growth dynamics tracking, we compared the biofilm initiation by saliva inoculum from the same pooled source but processed in two different ways: (i) the native inoculum, which contained intact communities of single cells and aggregates, and (ii) the dispersed inoculum, which was sonicated to disperse the natural aggregates without impacting bacterial viability. Our results showed that colonizing units in the dispersed group, comprised of mostly single cells and smaller aggregates, grew minimally with most of the cells remaining static, whereas the native community showed more active growth forming enlarged structures (Fig. 5a1). Furthermore, the biofilm initiating from the native inoculum displayed highly heterogeneous features, including peak-like architectures and a diverse range of thicknesses (Fig. 5a2, top, and b). In sharp contrast, the biofilm originating from the dispersed inoculum showed smaller and thinner structures that were like those in the dispersed colonizing community (Fig. 5a2, bottom, and b). Moreover, we found that bacteria naturally attached to human epithelial cells (host cell-microbial aggregates) can also grow faster than bacteria alone and proliferate rapidly to form large biofilm structures (Movie S2, Fig. S6). Hence, our results suggest that aggregated bacteria in the native state act as growth nuclei and play a pivotal role in the initiation and development of spatial heterogeneity of the biofilm community.

10.1128/mbio.00131-22.2 FIG S2 Contamination control and replicates of the microbiome experiments. Samples as contamination control correspond to sterile water used for biofilm collection (water) and cell-free saliva used as culture medium for biofilm growth (CLS). Principal correspondence analysis using Jaccard index for presence/absence of bacteria with study samples and control contamination samples are included. Download FIG S2, PDF file, 0.1 MB. Copyright © 2022 Simon-Soro and Ren et al. 2022 Simon-Soro and Ren et al. https://creativecommons.org/licenses/by/4.0/ This content is distributed under the terms of the Creative Commons Attribution 4.0 International license.

10.1128/mbio.00131-22.6 FIG S6 Microbial aggregates naturally attached to host cells grow faster than microbial aggregates alone. The inoculum (saliva) was either used untreated (host cell-microbial aggregates) or briefly sonicated to release bacteria from the epithelial cells (microbial aggregates). After inoculation, microbial growth in each group was tracked very 10 min for 12 h using bright-field imaging on a BioFlux microfluidics instrument. Biofilm formation was quantified as the percentage of biofilm coverage over time. Open symbols represent host cell-microbial aggregates; filled symbols correspond to microbial aggregates. Download FIG S6, PDF file, 0.1 MB. Copyright © 2022 Simon-Soro and Ren et al. 2022 Simon-Soro and Ren et al. https://creativecommons.org/licenses/by/4.0/ This content is distributed under the terms of the Creative Commons Attribution 4.0 International license.

### Taxonomic scale of native and dispersed communities.

Saliva, as a source for the biofilm formation, harbors hundreds of bacterial taxa (29). Here, we analyzed the microbial composition in samples from the native and dispersed communities, as shown in Fig. 5a. We collected different samples in a sequential manner: inoculum (baseline), binding (initial colonizing units), and 6-h biofilm. Samples for quality control and reproducibility check were also included (Fig. S2). We found that dispersion of the aggregates did not influence the types of bacteria that were attached to the HA surface, showing alpha and beta diversity differences were related to different time points but not between native and dispersed states (*P* *< *0.001) (Fig. 5c). Furthermore, we analyzed the relative abundance of bacterial genera at the three stages (inoculum, binding, and biofilm) (Fig. 5d). Notably, we detected 155 bacterial taxa bound to the HA from the 203 ASV (16S amplicon sequence variant) found in human saliva inoculum, showing that saliva richness is represented during biofilm initiation. After initial biofilm growth, the microbiota was reduced to 53 taxa, which was enriched by oxygen-tolerant bacteria compatible with the aerobic environment dominating biofilm initiation under our experimental conditions. Altogether, we found a highly diverse colonizing community, suggesting that microbial attachment is not exclusive to single cells or specific species but also encompasses a wide range of microbial aggregates containing a mixture of different species. Neither sequential nor orderly succession of selective binding of specific species was observed. Rather, the aggregates bound on the surface proliferate rapidly and expand tridimensionally, modulating population growth dynamics, spatial organization, and community scaffolding.

## DISCUSSION

**FIG 6:**
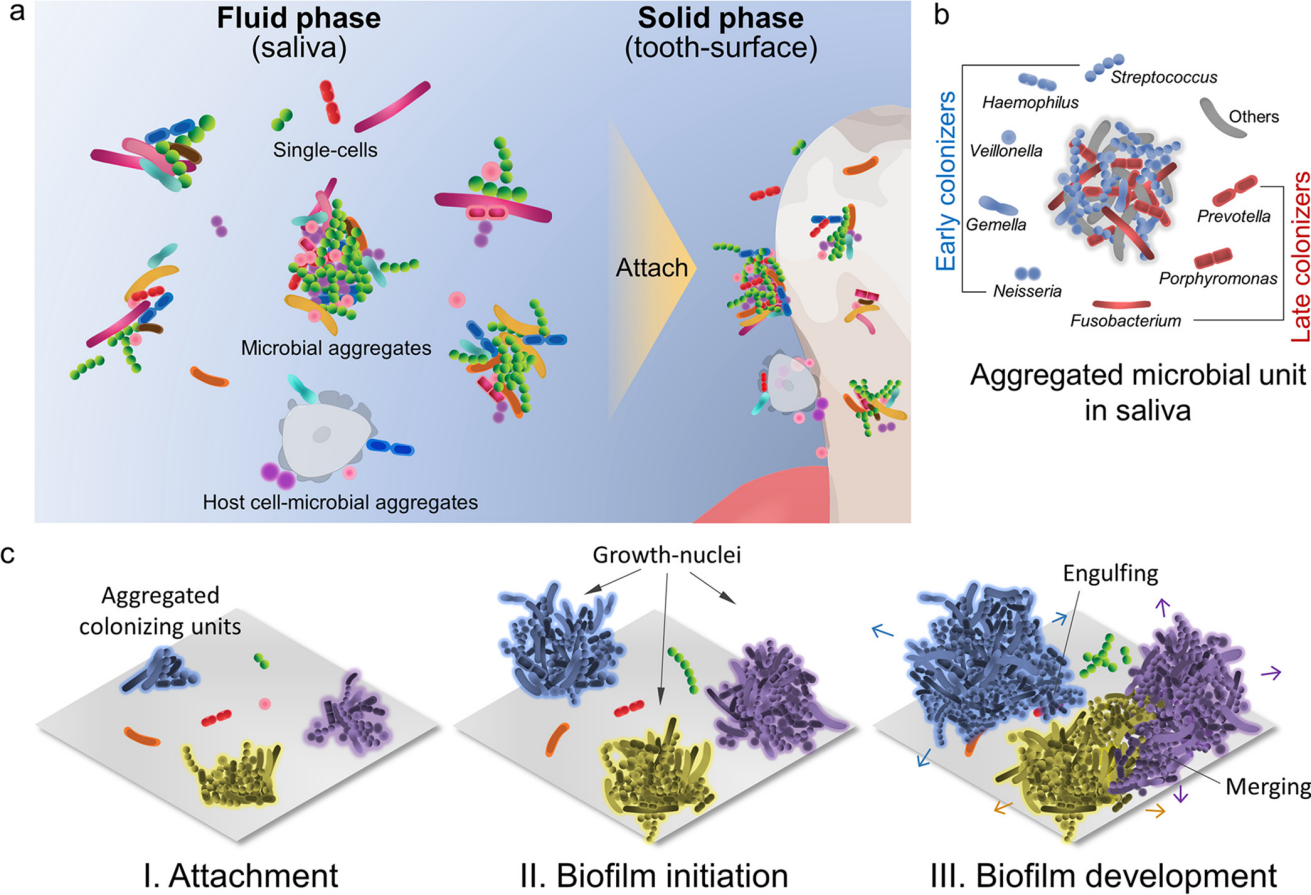
Schematic diagram of microbial aggregates as growth nuclei building the oral biofilm. (a) Saliva, the fluid source of microbes for dental biofilm, harbors structured aggregates (>90% of microorganisms are found in aggregated form) in addition to free-living bacterial cells (fluid phase). Notably, bacteria are also found coadhered with human epithelial cells, forming a highly complex and structurally diverse community. Both aggregates and single cells can bind to the tooth surface (solid phase). (b) Microbial aggregates in saliva harbor different bacterial taxa, including those traditionally considered early and late colonizers. Thus, the polymicrobial community can adhere to the surface collectively as structured colonizing units without an orderly attachment in a sequential fashion. (c) Microbial aggregates bound to the tooth surface act as growth nuclei that grow more actively and faster than the attached single cells, which stay static or grow slower. Actively growing aggregates expand tridimensionally, often engulfing the single cells and merging with other active growers to build the oral biofilm, forming spatially and compositionally heterogeneous superstructures.

Our findings reveal how naturally formed aggregates in the human oral environment mediate biofilm formation at different spatial, temporal, and phylogenetic scales. We show four key features, as summarized in Fig. 6. (i) Saliva, the source of microbes, across different subjects and timescales is dominated by polymicrobial aggregates harboring different species together in addition to free-living bacterial cells. Notably, bacteria are also found coadhered with human epithelial cells, indicating a more complex and structurally diverse community than previously described (7). (ii) Bacteria that traditionally have been considered early and late colonizers are found together in the adhering aggregates and in early formed human plaque samples, indicating that they bind concomitantly to the surface as a colonizing unit. (iii) Microbial aggregates bound to the enamel surface act as growth nuclei that grow more actively and faster than the attached single cells, which stay mostly static. When present, human epithelial cells harboring bacterial populations also appear to form nuclei for biofilm growth. (iv) Actively growing aggregates expand tridimensionally, often engulfing the static cells and merging with active growers, forming spatially heterogeneous superstructures but with reduced microbial diversity. These findings provide new perspectives for host-associated biofilm initiation and development that supports the highly clustered yet spatially and taxonomically heterogeneous communities found in clinical samples of intact dental biofilms (1–4).

The current paradigm for biofilm formation encompasses initial binding of single cells and sequential species-specific interactions mediated by adhesin-receptor attachment to the oral surfaces and interspecies coadhesion (5, 7, 15, 16, 30, 31). This well-established ecological succession model describes that early colonizing species first bind to the pellicle and form the basis for subsequent attachment of intermediate and late colonizers (7). *In vitro* systems using pure cultures of selected bacterial species in a planktonic state have been used with or without saliva, providing support for the sequential biofilm community assembly concept (16, 32). While such interactions have been demonstrated experimentally, we propose that, in addition to single-cell events, natural aggregated mixed communities can also colonize tooth surfaces in active form, and these preformed consortia have advantages over the single-cell counterparts under our experimental conditions. It is noteworthy that the current fluid-to-biofilm model has limitations to fully recapitulate the continuous microbial accretion from saliva *in vivo*. This can be implemented in future modifications of our system to better mimic the physiological biofilm growth dynamics.

Although the ecological succession model does not contradict that early colonizers could interact later with established biostructures or that late colonizers can be found in the early formed biofilms, our findings provide an alternative strategy utilized by human-derived microbial aggregates to colonize and build biofilms. This model would provide an explanation for the hedgehog or rotund structures found in the human dental plaque (1, 4) whereby bacteria are radially distributed with streptococci (traditionally considered early colonizers) located at the outermost layer. The aggregated nuclei formed by a mixed community could modify the microenvironment (e.g., reduce the oxygen levels) as it grows in an outward fashion, where aerobic and facultative anaerobic bacteria would preferentially occupy peripheral positions, while creating suitable conditions for late growers in the inner layers as the biofilm develops. Future studies could explore whether these biofilm structures are a consequence of these initial buds of growth.

The exact reasons why initial aggregated colonizers grow faster than the single cells are unclear when using saliva as the sole nutrient source. It is possible that such nutrient-limiting conditions are favorable for the aggregated community survival. Whether the inclusion of exogenous substrates (such as dietary sugars) or host-derived factors (such as inflammation) alter the dynamics of aggregated community growth or favors single-cell growth needs further elucidation. Furthermore, microbial species interact through specific associations that may have a nutritional, structural, or metabolic codependency during biofilm development (33, 34). How such interactions occur at the point of aggregated community binding to initial growth development needs further investigation. Moreover, it is an intriguing prospect that not all cells in the aggregated community are (equally) metabolically active, possibly harboring dormant cells that can become activated as it grows. Recent studies indicate that two neighboring communities display metabolic codependence or competitive interactions depending on the nutrient availability and bacterial composition (26, 35). Further investigations could also explore whether surface-attached single cells in close proximity to aggregated communities behave differently from those without aggregates nearby. Conversely, the findings on host epithelial cell-associated microbial communities open intriguing possibilities, suggesting a host-provided nutritional and scaffolding role contributing to community development on dental surfaces.

In summary, the data presented here point toward an alternative model (Fig. 6) whereby preformed consortia naturally present in human saliva govern biofilm initiation and microbial growth dynamics without specific taxonomic order or cell-by-cell succession to mediate spatial and community heterogeneity. This concept may also have therapeutic implications. For instance, the findings that most bacteria in saliva are in aggregated form could hamper the binding of salivary antibodies, reducing the efficacy of immune recognition and opsonization. This could explain the lack of success in developing active and passive immunization strategies against oral diseases (20). Likewise, most of the therapeutic or antifouling strategies are based on sequential single-cell binding, which may not target the aggregate binding and further biofilm development. In addition, these complex assemblages may already have a matrix in place that could protect them from the immune response and hinder the diffusion of antimicrobials (14). The proposed model may be extended to polymicrobial biofilms outside the oral cavity where aggregated consortia are commonly found, such as in other biological fluids or aquatic ecosystems (14), which could similarly enhance surface colonization and growth to cause biofilm-associated infections and environmental biofouling.

## MATERIALS AND METHODS

### Sample collection.

For flow cytometry, unstimulated saliva was collected from healthy volunteers (aged 20 to 40 years) at different time points after toothbrushing and processed immediately for analysis. For fluid-to-biofilm experiments, unstimulated saliva was collected from healthy individuals and pooled into a single source. Food debris and human cells were removed by brief centrifugation as detailed previously (36), and the processed saliva was used as the inoculum; some attached bacterial cells can be removed during this step. A saliva-based culture medium was prepared by pooling stimulated saliva from the same donors and filter sterilized. In addition, dental plaque samples from healthy volunteers were collected at 360 min after toothbrushing to assess the microbiota in the early formed biofilm. Details are provided in Text S1 in the supplemental material. All protocols were reviewed and approved by the ethical committee of Valencian Health Authority, Spain (BIO2015-68711-R), and the University of Pennsylvania’s Research Subject Committee (818549).

### Imaging analysis of microbial structures in saliva.

Superresolution confocal microscopy, field-emission scanning electron microscopy (FESEM), and transmission electron microscopy (TEM) were used to characterize the microbial structures in saliva. For confocal imaging, pooled unstimulated saliva was stained with 0.1 μM Syto9 (Molecular Probes). Immediately before imaging, saliva was pipetted onto presolidified agarose (1%) on a glass slide followed by a cover slip to immobilize the salivary microbes while preserving their native structure. This method did not cause aggregation of planktonic bacterial strains either alone or mixed. Confocal imaging was performed using a 40× water immersion objective on a Zeiss LSM800 microscope with Airyscan (37). FESEM and TEM imaging was performed using Zeiss FESEM UltraPlus 5 kV and JEOL/JEM1011-100kV.

### Sorting of salivary microbial structures by flow cytometry.

Salivary samples were prepared for fluorescence-activated cell sorting by following our previously reported protocol (20). The small (<3 μm) and large (>3 μm) fractions (based on size clustering by flow cytometry) were separated by the sorter and frozen at −80°C for further analysis. Details on calibration, validation, and sorting criteria can be found in Text S1 in the supplemental material.

10.1128/mbio.00131-22.10 TEXT S1 A detailed description of the methods used in the study. Download Text S1, PDF file, 0.1 MB. Copyright © 2022 Simon-Soro and Ren et al. 2022 Simon-Soro and Ren et al. https://creativecommons.org/licenses/by/4.0/ This content is distributed under the terms of the Creative Commons Attribution 4.0 International license.

### Bacterial composition in sorted fractions of saliva.

Bacterial DNA was extracted from the small and large fractions by a combination of physical and chemical cell lysis. Samples were first physically lysed by bead beating (Tissuelyzer II; Qiagen) followed by chemical lysis using lysozyme (37°C for 30 min) and DNA extraction using the RNA/DNA MasterPure extraction kit (Epicentre). The 16S rRNA genes (regions V1 to V4) then were amplified. Amplicons were sequenced using the 454 GS-FLX pyrosequencer (Titanium chemistry; Roche). To analyze 16S RNA gene sequences, we used QIIME2 v19.4 (38). We trimmed the pyrosequences using Cutadapt, obtaining sequences 750 bp in length with a quality of >25 (39). We obtained amplicon sequence variants (ASV) for analysis through DADA2 denoise-pyro, specifically designed for pyrosequencing sequences (40). We then obtained taxonomic assignments based on the GreenGenes 16S rRNA gene database v.13_8, trained using scikit-learn 0.20.2 (41).

### Active microbiota composition in dental plaque.

RNA was extracted after a combination of physical and chemical cell lysis as described above. RNA extraction and DNase treatment were performed using the RNA/DNA MasterPure extraction kit (Epicentre). Single-stranded cDNA was constructed with the high-capacity cDNA reverse transcription kit (Applied Biosystems) in 20-μl reaction mixtures (42). To assess the 16S rRNA gene region, we amplified regions V1 to V4 from the single-stranded cDNA (43). Amplicons were sequenced in the GS-FLX pyrosequencer (Roche) with Titanium-plus chemistry. For data analysis, only reads longer than 400 bp were selected to increase accuracy in taxonomic assignment.

### Fluid-to-biofilm model and *ex vivo* biofilm dynamics.

We developed a fluid-to-biofilm model to investigate the dynamics of the biofilm development from saliva on a tooth-mimetic surface using our continuous flow-cell labeling and confocal imaging protocol (26). Briefly, a saliva-coated hydroxyapatite disk was placed in a vertical position to mimic the smooth surfaces of human teeth and immersed in the saliva inoculum to allow bacterial binding. After a 60-min incubation, which was predetermined to yield the optimum binding (see Fig. S1 in the supplemental material), the disk with the initial colonizing community (including single cells and aggregates) was prestained with 0.1 μM Syto9. The disk then was gently washed to remove loosely bound bacteria and aseptically transferred into a flow-cell microfluidics device (BioSurface Technologies) for biofilm development analysis via time-lapsed confocal imaging. A saliva-based culture medium containing 250 nM Syto9 was continuously provided (100 μL/min) to allow continuous labeling of the growing biofilm. Time-lapsed confocal acquisition was performed every 30 min using a 40× water immersion objective on a Zeiss LSM800 with Airyscan. Experimental details can be found in Text S1 in the supplemental material.

### Metagenomics for the fluid and solid phases during microbial binding.

Saliva inoculum and binding samples were aliquoted into PowerBead Pro tubes and frozen at −80°C until analysis. DNA was extracted using the DNeasy PowerSoil kit (Qiagen) and quantified with the Quant-iT PicoGreen assay kit (Molecular Probes). Shotgun libraries were generated from 0.5 ng DNA using the Nextera XT library prep kit (Illumina), and libraries were sequenced on an Illumina HiSeq 2500 in high-output mode to produce paired-end 125-bp sequence reads. Extraction blanks and nucleic acid-free water were processed along with experimental samples to empirically assess environmental and reagent contamination (Fig. S2). A laboratory-generated mock community consisting of DNA from Vibrio campbellii and lambda phage was included as a positive sequencing control. For bioinformatics processing and analysis, shotgun metagenomic data were analyzed using Sunbeam (44). The abundance of bacteria was estimated using Kraken (45). Diversity within samples was assessed by the number of operational taxonomic units at a rarefaction level of 1,000 sequences and the Shannon index.

### Microbiome in fluid-to-biofilm model dynamics.

To assess the microbial composition dynamics in the fluid-to-biofilm model, samples were collected from the native and the dispersed groups at three different stages (inoculum, binding, and biofilm), as detailed in Text S1. DNA was extracted, and PCR amplification (V1-V2 region) of the 16S rRNA gene was performed by following previously reported methods (46). The library was sequenced to obtain 2 × 250-bp paired-end reads using MiSeq (Illumina). To analyze 16S RNA gene sequences, we used QIIME2 v19.4 (38). We obtained taxonomic assignments based on the GreenGenes 16S rRNA database v.13_8 (42) and ASV analysis of shared and unique bacterial taxa through DADA2 (40).

### Biofilm formation from host cell-microbial aggregates using BioFlux.

Biofilm formation from host cell-microbial aggregates and microbial aggregates was tracked using a BioFlux system (Fluxion Biosciences) and fluorescence microscopy as described by Hoogenkamp et al. (47). Briefly, whole saliva samples containing host cell-microbial aggregates were incubated in a 48-well BioFlux plate to allow aggregate binding. Biofilm formation then was monitored via time-lapse imaging using a 20× objective as detailed in Text S1.

### Image processing and quantitative analysis.

Computational processing and quantitative imaging analysis were performed using BiofilmQ, an image analysis platform optimized for biofilm structure and formation dynamics (28). Growth dynamics of individual colonized units (single cells or aggregates) was assessed using the cube tracking algorithm that allows each colonizing unit to be tracked individually and spatiotemporally. Only the single cells/aggregates that were attached and remained bound throughout the experiment were included in the analysis. Details are provided in Text S1.

### Statistical analysis.

R environment (version 4.0.3) was used for statistical analysis. The level of statistical significance was 0.05. The library ape was used for the principal coordinate analysis for beta diversity based on Jaccard distance (48). Wilcoxon rank sum test was used to compare the cell counts in small/large fractions in flow cytometry, the biovolume fold change of single cells/aggregates during biofilm growth, and the richness (alpha diversity) at different stages (inoculum, binding, and biofilm). One-way analysis of variance with Dunnett’s multiple-comparison test was used to detect differences between groups when analyzing the surface coverage by microorganisms in saliva inoculum.

### Data availability.

16S rRNA sequencing data for flow cytometry, fluid-to-biofilm *in vitro* experiments, and *in vivo* dental plaque samples were deposited in NCBI BioProject under accession number PRJNA739848. Shotgun metagenomics sequencing data were deposited in NCBI BioProject under accession number PRJNA800381.

10.1128/mbio.00131-22.9 MOVIE S2 Biofilm formation using the BioFlux microfluidics system. Whole saliva samples containing host cell-microbial aggregates were used, and the biofilm formation was monitored every 10 min for 12 h on a BioFlux instrument. Microbial aggregates are seen as nuclei for biofilm growth. Download Movie S2, MOV file, 5.9 MB. Copyright © 2022 Simon-Soro and Ren et al. 2022 Simon-Soro and Ren et al. https://creativecommons.org/licenses/by/4.0/ This content is distributed under the terms of the Creative Commons Attribution 4.0 International license.
