# Supplementary material for: Polymicrobial Aggregates in Human Saliva Build the Oral Biofilm
Source: mBio. 2022 Feb 22;13(1):e00131-22. doi: 10.1128/mbio.00131-22 (PMC8903893; doi:10.1128/mbio.00131-22)
Supplement: TABLE S1 [file mbio.00131-22-st001.pdf]

Supplemental Table. Cell event counts in small/large fractions of saliva from human subjects. Data from samples collected at different time points (30 min, 90 min, 150 min, and 360 min after toothbrushing) are included. Data are presented as Mean, minimum (Min), maximum (Max), standard deviation (SD) and percentage.

Subject	Cluster Structure	Mean	Min	Max	SD	Percentage
PGN01	Small	53273.40	5030	91512	40373.44	3.49
PGN01	Large	1472242.80	412366	362559	1344947.83	96.51
PGN02	Small	134133.75	87407	211164	55430.85	5.31
PGN02	Large	2391592.75	212790	5348572	2322464.10	94.69
PGN03	Small	40077.75	24294	64593	19172.71	0.24
PGN03	Large	16515347.00	3842045	25847817	10507207.03	99.76
PGN05	Small	66860.50	35139	98882	26031.01	1.12
PGN05	Large	5892810.50	2319764	8793401	3270117.00	98.88
PGN06	Small	67722.75	42845	101002	24719.77	3.52
PGN06	Large	1854648.75	873343	4119465	1535786.39	96.48
PGN07	Small	53031.25	26771	87928	25913.69	5.38
PGN07	Large	932778.00	516495	1541281	450226.31	94.62
PGN08	Small	61469.75	25380	97259	29866.83	3.05
PGN08	Large	1957029.75	175719	7074026	3411903.32	96.95
PGN010	Small	186164.83	145539	290300	55124.56	18.91
PGN010	Large	798545.67	586873	1131931	205253.36	81.09
