# Supplementary material for: Polymicrobial Aggregates in Human Saliva Build the Oral Biofilm
Source: mBio. 2022 Feb 22;13(1):e00131-22. doi: 10.1128/mbio.00131-22 (PMC8903893; doi:10.1128/mbio.00131-22)
Supplement: FIG S3 [file mbio.00131-22-sf003.pdf]

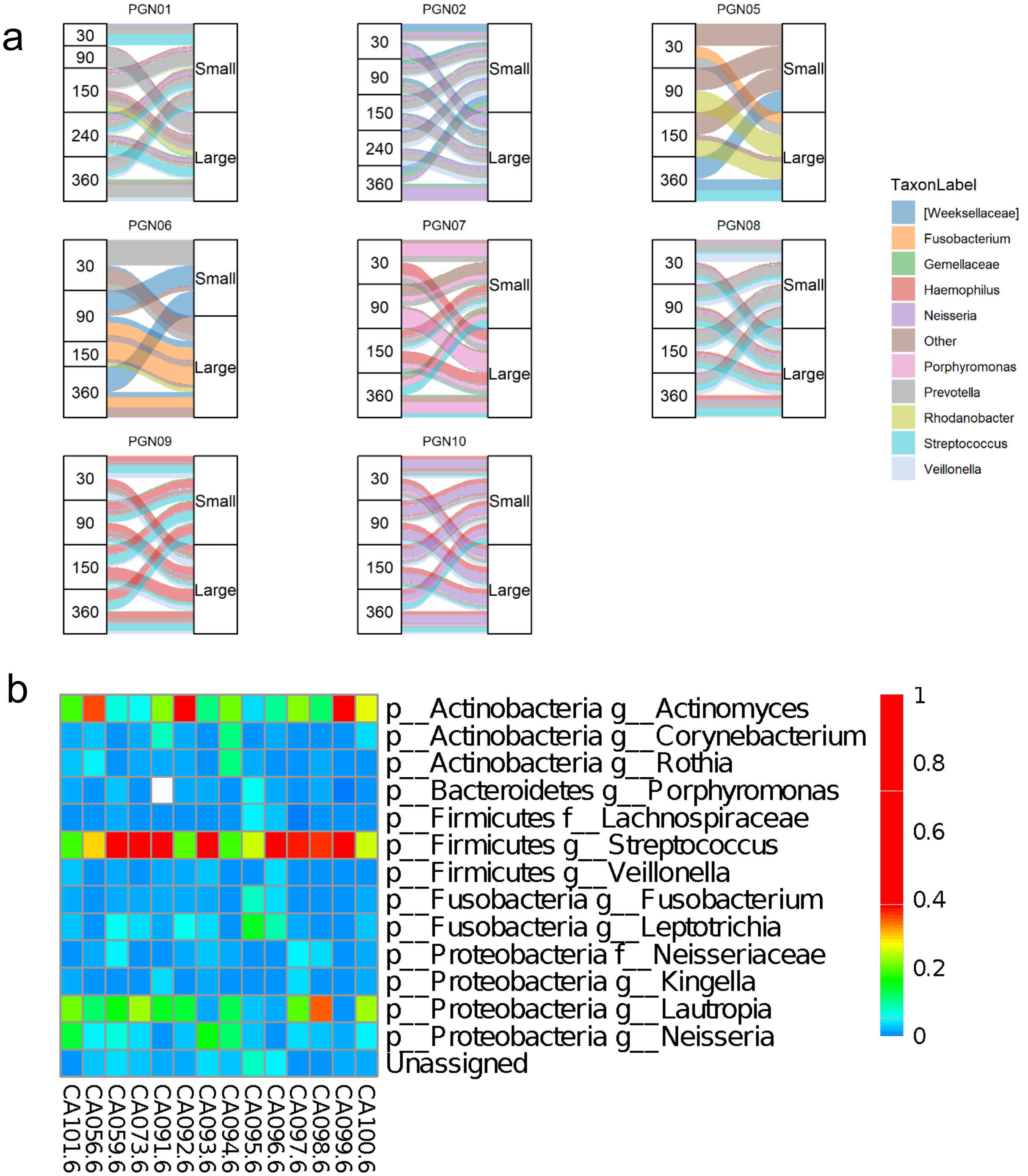

Supplemental Fig. 3. (a) Subject specific microbial abundances by saliva fraction. Alluvial plot visualizes the bacterial abundance (flow line thickness) found on small and large fractions in sorted saliva samples for each subject. Samples were taking at 30, 90, 150 and 360 minutes after toothbrushing. **(b) Active bacteria in supragingival dental plaque (360 min after toothbrushing).** Bacterial composition was determined by high-throughput sequencing of the 16S rRNA gene after RNA extraction. Heatmap shows the proportion of the main active bacteria in the dental plaque metatranscriptome at 360 min post-toothbrushing on 14 subjects.
