# Supplementary material for: Polymicrobial Aggregates in Human Saliva Build the Oral Biofilm
Source: mBio. 2022 Feb 22;13(1):e00131-22. doi: 10.1128/mbio.00131-22 (PMC8903893; doi:10.1128/mbio.00131-22)
Supplement: FIG S1 [file mbio.00131-22-sf001.pdf]

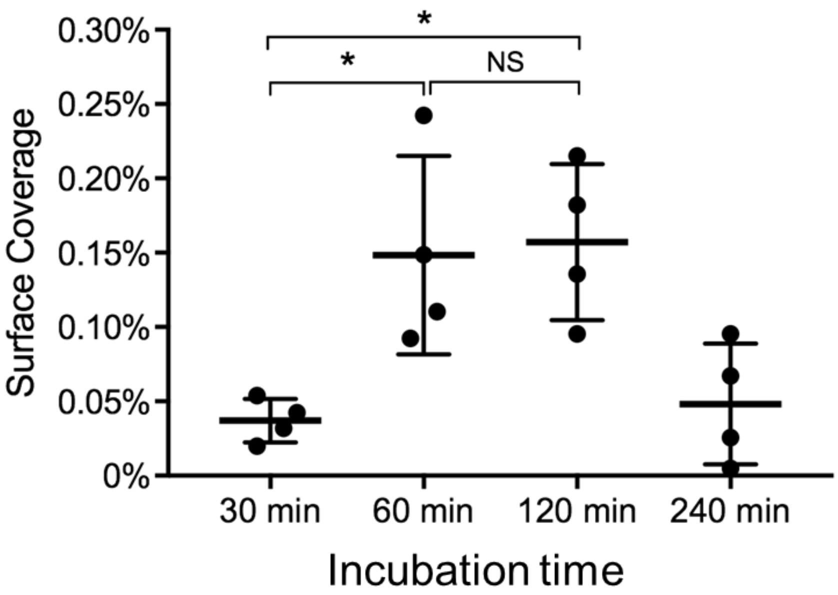

Supplemental Fig. 1. Surface coverage by microorganisms in biofilms formed from saliva inoculum after 30 min, 60 min, 120 min, and 240 min. Surface coverage by the colonizing microbial community was determined using confocal microscopy and computational image analysis with BiofilmQ. Maximum binding with the highest coverage was reached after 60 min of incubation which was used for further experiments. *, $P<0.05$, one-way analysis of variance (ANOVA) with Dunnett's multiple comparisons test. NS, not significantly different.
