# Supplementary material for: Polymicrobial Aggregates in Human Saliva Build the Oral Biofilm
Source: mBio. 2022 Feb 22;13(1):e00131-22. doi: 10.1128/mbio.00131-22 (PMC8903893; doi:10.1128/mbio.00131-22)
Supplement: FIG S4 [file mbio.00131-22-sf004.pdf]

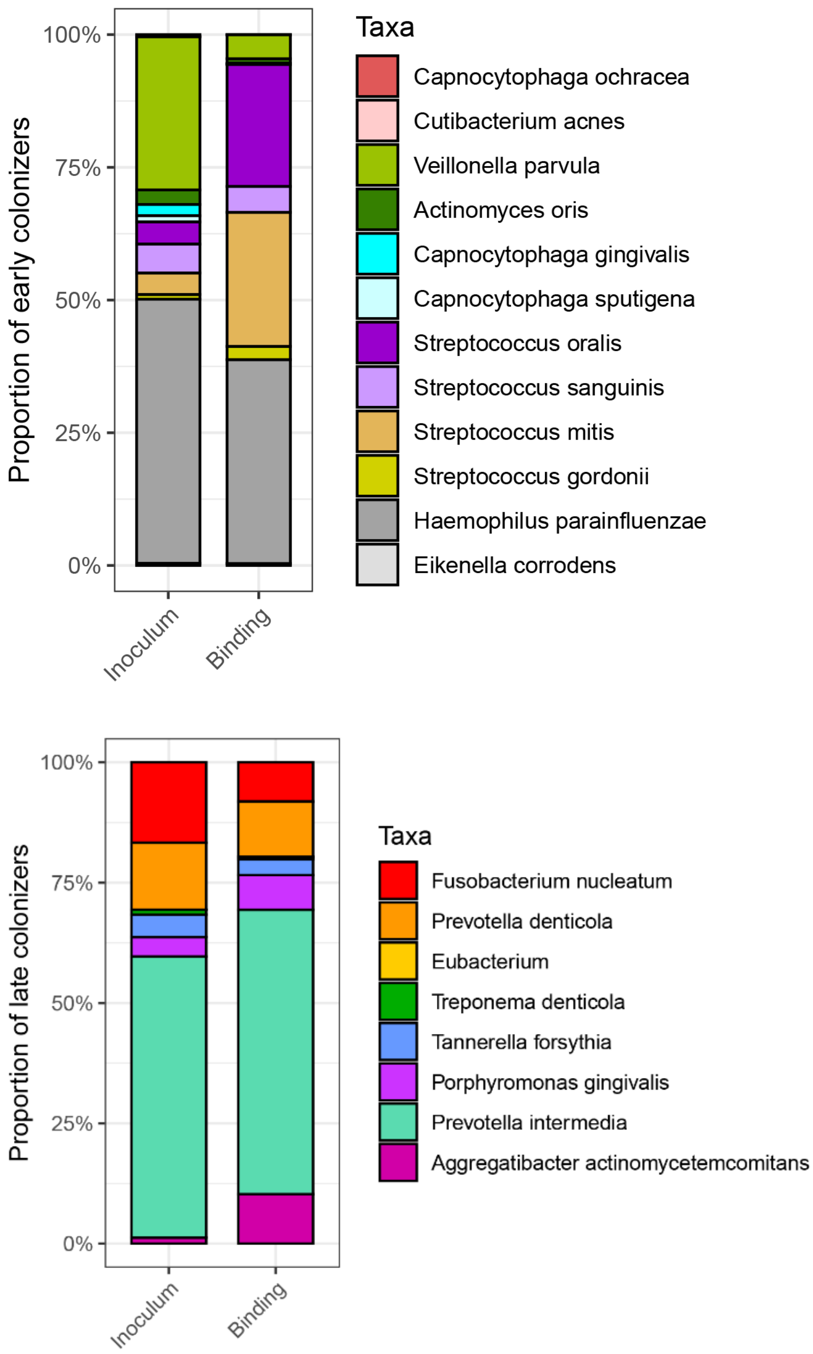


**Supplemental Fig. 4. Microbial species in the saliva inoculum and at binding.** The abundance of each species on the Y-axis is relative to the total amount of either early (top panel) or late colonizers (bottom panel). Early colonizers detected include *Streptococcus, Haemophilus, Capnocythophaga, Actinomyces, and Veillonella*, whereas species that are traditionally considered late colonizers such as *Prevotella, Treponema, Aggregatibacter*, and *Porphyromonas* were also found in the initial colonizing community, suggesting mixed-species co-colonization during the binding process. *Fusobacterium nucleatum*, a bridging organism, was also found in this initial colonizing community.
