# Supplementary material for: Polymicrobial Aggregates in Human Saliva Build the Oral Biofilm
Source: mBio. 2022 Feb 22;13(1):e00131-22. doi: 10.1128/mbio.00131-22 (PMC8903893; doi:10.1128/mbio.00131-22)
Supplement: FIG S5 [file mbio.00131-22-sf005.pdf]

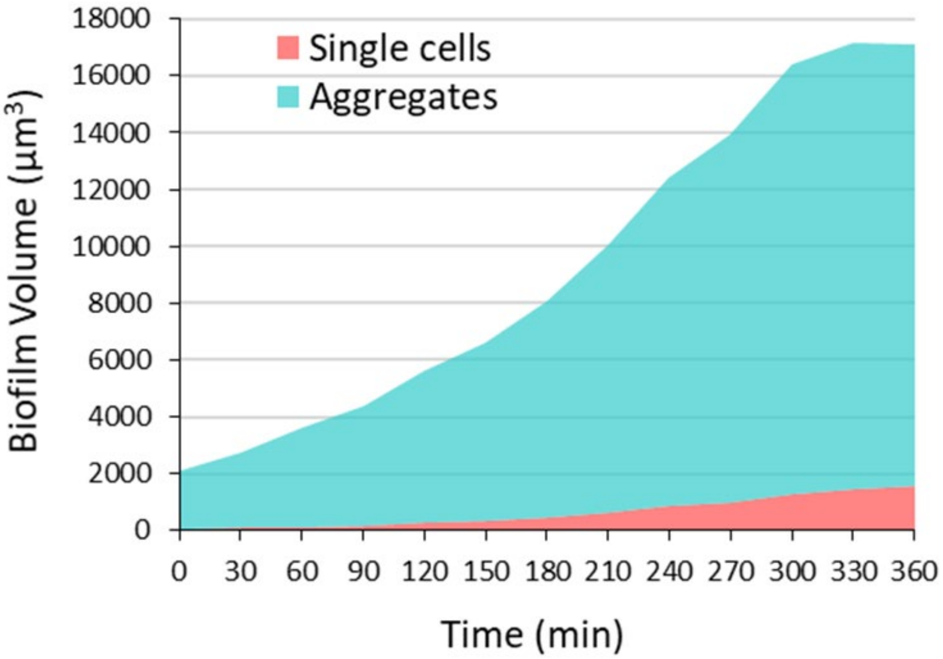

Supplemental Fig. 5. Total biovolume of single-cell/aggregate-derived biofilm. Time-resolved biofilm volume originated from colonizing units in different sub-groups ("single-cells" and "aggregates") on a surface area of 159.73 × 159.73 μm^2. Saliva-derived biofilm growth was followed by time-lapsed confocal imaging in the Fluid-to-Biofilm system. Data were computationally analyzed using BiofilmQ. The total biovolume from an aggregate origin (in blue) increased over time accounting for >90% of the total amount at the endpoint (~17000 μm^3). In contrast, single-cell derived biovolume (in pink) remained low resulting in less than 1,500 μm^3 at 360 min.
