# Supplementary material for: Polymicrobial Aggregates in Human Saliva Build the Oral Biofilm
Source: mBio. 2022 Feb 22;13(1):e00131-22. doi: 10.1128/mbio.00131-22 (PMC8903893; doi:10.1128/mbio.00131-22)
Supplement: FIG S2 [file mbio.00131-22-sf002.pdf]

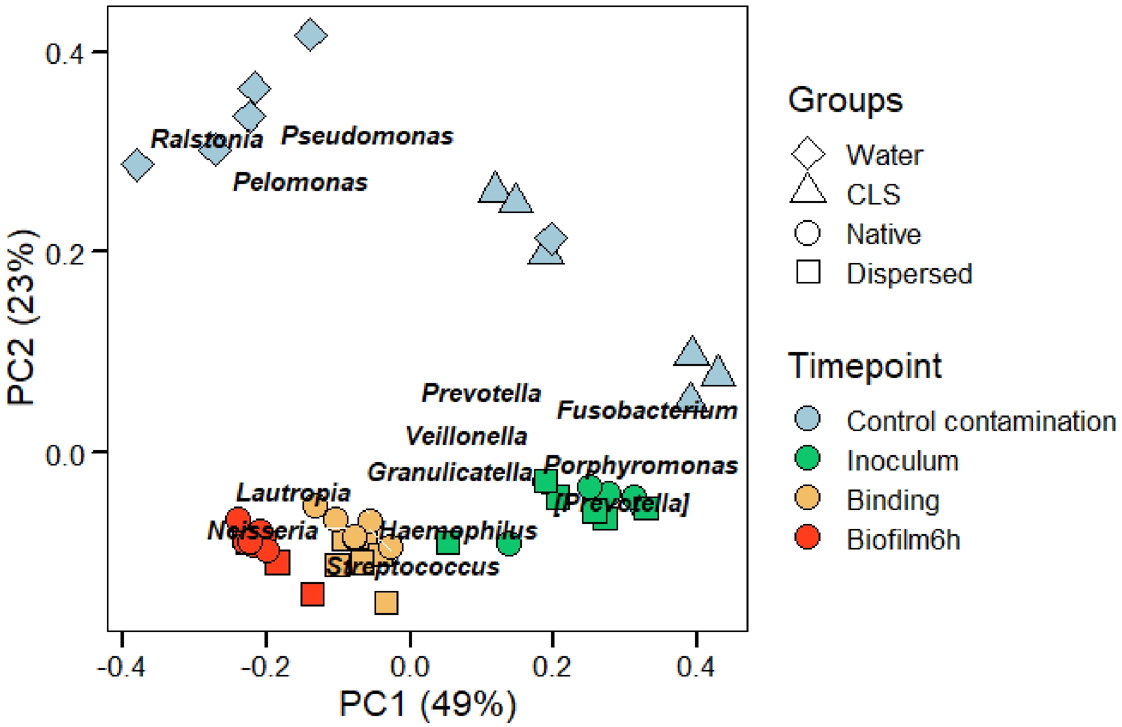

Supplemental Fig. 2. Contamination control and replicates of the microbiome experiments. Samples as contamination control correspond to sterile water used for biofilm collection (water) and cell-free saliva used as culture medium for biofilm growth (CLS). Principal correspondence analysis using Jaccard index for presence/absence of bacteria with study samples and control contamination samples are included.
