# Supplementary material for: Polymicrobial Aggregates in Human Saliva Build the Oral Biofilm
Source: mBio. 2022 Feb 22;13(1):e00131-22. doi: 10.1128/mbio.00131-22 (PMC8903893; doi:10.1128/mbio.00131-22)
Supplement: FIG S6 [file mbio.00131-22-sf006.pdf]

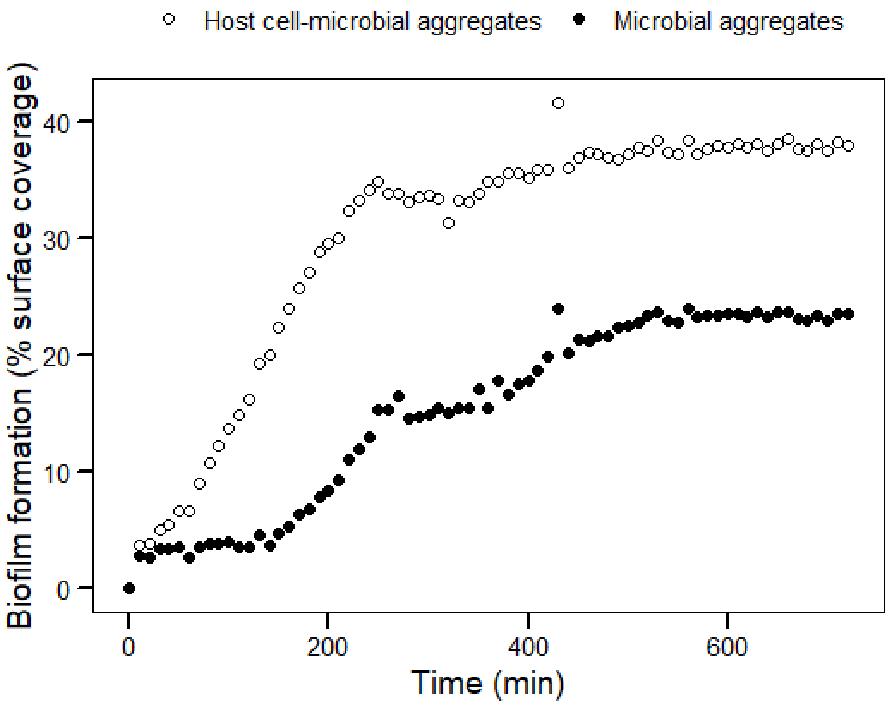

Supplemental Fig. 6. Microbial aggregates naturally attached to host cells grow faster than microbial aggregates alone. The inoculum (saliva) was either used untreated (host cell-microbial aggregates) or briefly sonicated to release bacteria from the epithelial cells (microbial aggregates). After inoculation, microbial growth in each group was tracked very 10 min for 12 h using brightfield imaging on a Bioflux microfluidics instrument. Biofilm formation was quantified as the percentage of biofilm coverage over time. Open symbols represent host cell-microbial aggregates; filled symbols correspond to microbial aggregates.
