# Supplementary material for: Polymicrobial Aggregates in Human Saliva Build the Oral Biofilm
Source: mBio. 2022 Feb 22;13(1):e00131-22. doi: 10.1128/mbio.00131-22 (PMC8903893; doi:10.1128/mbio.00131-22)
Supplement: TEXT S1 [file mbio.00131-22-s0001.pdf]

TEXT S1

Supplemental Methods for
Polymicrobial Aggregates in Human Saliva Build the Oral Biofilm

Sample collection

For flow cytometry, unstimulated saliva was collected from 8 healthy volunteers (aged 20-40 years) with informed consent. Each subject had at least 28 teeth from permanent dentition with no active dental caries, periodontal disease or systemic conditions. No antibiotics, antiseptics or antifungals were used in 90 days prior to the sample collection. Saliva samples were collected 30 min (at 9 AM), 90min, 150 min, 240 min and 360 min after toothbrushing. A total volume of 3 mL saliva was collected from each donor at each time point. Samples were processed immediately for flow cytometry analysis.

For the fluid-to-biofilm experiment, both unstimulated and stimulated saliva was collected from at least three healthy individuals (same criteria as stated above), respectively. The unstimulated saliva was collected from each donor and was pooled into a single source before brief centrifugation (2,600g for 10 min at 4 ºC) to remove food debris and human cells (1). This processing step only removes large particles, mostly human cells with minimal amount of attached bacterial cells as checked under the microscope in pre- and post-processed samples. The saliva was then used as inoculum for the biofilm assays. For experiments comparing the native/dispersed inoculum, an aliquot from the same pooled unstimulated saliva source was further processed by brief probe sonication (10 s on, 10 s off for 3 cycles, 7-watt output) (Branson Sonifier 150; Branson Ultrasonics, Brookfield, CT, USA), which was pre-optimized to disperse the native aggregates without impacting bacterial viability. A saliva-based culture medium was prepared by pooling stimulated saliva from the same donors (by parafilm-chewing), centrifuging (5,500 x *g*, 4°C, 10 min) and filter-sterilization (polyethersulphone low-protein binding filter with 0.22-μm-diameter pore size; Millipore, Burlington, MA, USA).

During and after fluid-to-biofilm experiments, samples were taken for microbiome at different time points, and from both the native and the dispersed groups. Samples were collected at the following time points: (i) the inoculum, which was the pooled saliva sample after processing; (ii) binding, which was the initial colonizing community on hydroxyapatite disks after the 60-min binding; (iii) 360-min biofilm, which was the microbial community on hydroxyapatite disks after 360 min of growth. We validated the reproducibility of our fluid-to-biofilm model, showing low microbiome variability between independent experiments (Fig. S2). At least three independent experiments were performed.

To assess the active microbiota in the initial biofilm, we collected dental plaque samples from 14 healthy volunteers (7 males and 7 females, average age 34 ± 9.3 years; same criteria as above). Samples were collected at 360 min after toothbrushing using a sterile excavator spoon. Dental plaque was collected from both buccal and lingual smooth surfaces of all the teeth except the third molars. Then, samples from the first and third quadrants were pooled for each individual.

All subjects provided written informed consent. Experimental protocols were reviewed and approved by the ethical committee of Valencian Health Authority, Spain (approval number: BIO2015-68711-R) and the University of Pennsylvania's Research Subject Committee (approval number: 818549).

Sorting of salivary microbial structures by flow cytometry

Fluorescence-activated cell sorting was employed to separate the different microbial structures in saliva based on size. Samples in sterile saline solution were sorted with MoFlo XDP Cell Sorter (Beckman Coulter) using argon-blue laser (488 nm, 200 mW) and red laser (635 nm, 25 mW) as the light source. The flow cytometer was calibrated using fluorescent microspheres Flow-Check (10-µm diameter) and Flow-Set (3-µm diameter) (Beckman Coulter, Pasadena, CA, USA). Microbial structures in saliva were separated into 2 subpopulations based on the clustering of the flow cytometry data which were described as small (< 3 µm) and large (> 3 µm) fractions. To

quantify the number of individual cells in the large fraction, we sonicated the saline solution containing the large fraction for 30 seconds (Raypa VCI-50, Barcelona, Spain) to disperse the aggregates without breaking bacterial cells. Then, samples were filtered through 20-µm filters to remove human cells before being reanalyzed by flow cytometry. 1-second pulses of compressed air were applied, when necessary, to minimize the number of bacterial cells attached to the filter. The small fraction was not sonicated or filtered, but was directly analyzed with the flow cytometer for cell counting.

Fluid-to-biofilm model and *ex vivo* biofilm dynamics

We developed a fluid-to-biofilm model to investigate the dynamics of the biofilm development from saliva on a tooth-mimetic surface, based on our continuous flow-cell labelling and confocal imaging system optimized for oral biofilms (2). Biofilms were formed on a saliva-coated hydroxyapatite (2.7 ± 0.2 cm^2; Clarkson Chromatography Products, Inc., Williamsport, PA, USA) disk which served as a tooth enamel surrogate. The disks were placed in a vertical position using a custom-made holder to mimic the smooth surfaces of human teeth and were immersed in the saliva inoculum to allow bacterial binding. After 60 min of incubation (37 °C, 5% CO_2) which was pre-determined to yield the optimum binding (Fig. S1), the disk with the initial colonizing community was pertained with 0.1 µM Syto 9 (485/498 nm; Molecular Probes Inc., Eugene, OR) in 0.9% NaCl for 5 min at 37 °C. Then, the disk was gently washed to remove loosely bound bacteria and aseptically transferred into a flow-cell microfluidics device (BioSurface Technologies, Bozeman, MT, USA) for biofilm development analysis via time-lapsed confocal imaging. A saliva-based culture medium (as detailed in Sample Collection) was continuously provided in the microfluidic system by a peristaltic pump at a flow rate of 100 µL/min to mimic the natural nutrient condition of dental plaque in the oral cavity. The saliva-based medium was supplemented with 250 nM Syto 9 to allow continuous labelling of the growing biofilm. This concentration was pre-determined showing optimized bacterial labeling without any negative effects on cell growth. The time point denoted as t_0 corresponds to the time of the first confocal image acquisition before starting the flow and images were taken every 30 min after the start of the flow. Syto 9 was excited using a 488 nm laser. Confocal images (0.077-

µm pixel size and 0.37-µm Z step) were acquired at a 30-min interval using a 40× (numerical aperture = 1.2) water immersion objective mounted on a Zeiss LSM 800 upright confocal microscope with Airyscan. ImageJ FIJI (https://imagej.net/Fiji) was used for biofilm visualization.

Microbiome in fluid-to-biofilm model dynamics

To assess the microbial composition dynamics in the fluid-to-biofilm model, samples for microbiome analysis were collected from the native and the dispersed groups at three different stages (inoculum, binding, and biofilm) as detailed in Sample Collection. For the binding, samples were collected by bath sonicating the hydroxyapatite disk with adhered bacteria in 1 mL sterile phosphate-buffered saline (PBS) for 10 min. The biofilm samples were collected by swabbing using a flocked nylon swab (519C FLOQSwab, COPAN Diagnostics Inc. Murrieta, CA, USA) and eluting into sterile PBS. All samples were stored in -80°C until DNA extraction. Contamination control samples, including blank swabs, PBS, DNA-free water and reagent controls were collected. Also, mock DNA samples (positive controls) were included following the reported method (3). At least 3 independent experiments were conducted for each condition. We validated the reproducibility of our fluid-to-biofilm model, showing low microbiome variability between independent experiments (Fig. S2). DNA was extracted using the DNeasy PowerSoil kit (Qiagen, Valencia, CA, USA) according to the manufacturer's instruction within a class II laminar flow hood. PCR amplification of V1-V2 region of 16S rRNA gene was performed following previously reported methods (4). The library was sequenced to obtain 2 x 250 bp paired-end reads using the MiSeq (Illumina, San Diego, Ca, USA). To analyze 16S RNA gene sequences, we used QIIME2 v19.4 (5). We obtained taxonomic assignments based on GreenGenes 16S rRNA gene database v.13_8 (6). We obtained amplicon sequence variant (ASV) for analysis of shared and unique bacterial taxa through DADA2 (7).

Biofilm formation using the Bioflux

To analyze the role of the host cell-microbial aggregates in biofilm initiation, saliva was collected at two time points after tooth brushing, from a single, self-reported, healthy volunteer. This volunteer

was instructed to refrain from oral hygiene from eating for 16 h and drinking for 2 h before toothbrushing without toothpaste with a medium soft toothbrush (Colgate Palmolive, New York, NY, USA). Either 30 min or 6 h after brushing, whole stimulated saliva was collected by chewing on two gum pellets without active ingredients (Wrigley, Chicago, IL, USA) and aspirated into a non-chilled tube for further processing.

To remove adhered bacterial cells from the buccal cell and to disperse the salivary content the saliva was divided into four 1 mL aliquots of which two were sonicated, on ice, 30 times, 1 s, at 40 Hz (VC130 Ultrasonic processor, Sonics & Materials, Newtown, CT, USA). To analyze the role of the buccal cell-adhered and non-adhered bacteria, either 50 µL sonicated or non-sonicated saliva was back-flowed into phosphate buffered saline filled channels of a 48-well BioFlux plate (Fluxion Biosciences, San Francisco, CA, USA) and incubated for 1 h at 37°C to allow the salivary content to adhere to the glass surface.

To monitor biofilm formation, the plate was mounted on the stage of an inverted microscope after which brightfield images were acquired at three individual positions in multiple channels. Biofilm formation was initiated by flowing, artificial saliva medium supplemented with 50 mM PIPES at a flowrate of 0.5 dyne/cm^2 through the channels after the first image acquisition. Biofilm formation was followed in time for 12 h collecting images every 10 min using a 20× objective. Microscope and software details are described in detail by Hoogenkamp et al. 2015 (8). For each position, stacks were created using ImageJ software Version 1.48V (rsb.info.nih.gov/ij). Stacks containing buccal cells were analyzed to ensure buccal cells remained attached during the experiment. The remaining stacks were used for image analysis. In short, of each stack, the first image served as a reference point for buccal and bacterial cell-associated bacterial growth. To semi-quantify the extent of biofilm formation, within the same stack, grids of equal size were placed around attached buccal or bacterial cells. Subsequently, using the Time Series Analyser V3 plugin of the ImageJ software the decrease in average intensity was calculated in time and expressed as the percentage biofilm coverage in time. Each experiment was repeated at least twice using fresh saliva each time.

Image processing and quantitative analysis

Computational processing and quantitative imaging analysis were performed using BiofilmQ (https://drescherlab.org/data/biofilmQ/), an image analysis platform optimized for biofilms (9). The spatial distribution and growth of individual colonized units (single-cells or aggregates) were assessed using the cube tracking algorithm in BiofilmQ that allows each colonizing unit to be tracked individually and spatiotemporally. The raw image stacks containing 3-dimentional data of the microorganisms at each analyzed time point were time-stitched into a time series, thus resulting in a 4-dimentional (x, y, z, and time) dataset. The datasets were converted into BiofilmQ's TIF image format and the stage drift during time-lapse imaging was corrected using the image alignment function. After biofilm segmentation using an optimized threshold, we performed a cube-based object declumping that dissected a larger biofilm volume into small cubic volumes. This function allows further analysis of biofilm properties inside the biofilm volume with spatial resolution. Object parameters for each cube inside a biofilm cluster, e.g., local shape volume, local thickness and their geometrical coordinates were calculated. The biovolume was calculated using the binarized z-stacks. We further grouped all the initial colonizing units based on their biovolume (V): small clusters with $V \leq 10\ \mu m^3$ which represent the total biovolume of up to five cocci-like cells (defined as single cells) (2) and large clusters with $V > 10\ \mu m^3$ (defined as aggregates). The growth of individual colonized units was followed using the cube tracking algorithm in BiofilmQ. Biofilm structures (cubes) originating from the same initial colonizing units were assigned the same track identifier so that each cluster could be tracked individually. To eliminate the discrepancy of total growth time caused by bacteria detachment and new attachment, we applied a temporal filtering to only include those units that attached at the initial binding (t_0) and remained bound throughout the experiment (within 360 min) into our growth analysis.

References

1. Edlund A, Yang Y, Hall AP, Guo L, Lux R, He X, Nelson KE, Nealson KH, Yooseph S, Shi W, McLean JS. 2013. An *in vitro* biofilm model system maintaining a highly reproducible species and metabolic diversity approaching that of the human oral microbiome. Microbiome 1:25.

2. Paula AJ, Hwang G, Koo H. 2020. Dynamics of bacterial population growth in biofilms resemble spatial and structural aspects of urbanization. Nat Commun 11:1354.

3. Kim D, Hofstaedter CE, Zhao C, Mattei L, Tanes C, Clarke E, Lauder A, Sherrill-Mix S, Chehoud C, Kelsen J, Conrad M, Collman RG, Baldassano R, Bushman FD, Bittinger K. 2017. Optimizing methods and dodging pitfalls in microbiome research. Microbiome 5:52.

4. Liu Y, Naha PC, Hwang G, Kim D, Huang Y, Simon-Soro A, Jung H-I, Ren Z, Li Y, Gubara S, Alawi F, Zero D, Hara AT, Cormode DP, Koo H. 2018. Topical ferumoxytol nanoparticles disrupt biofilms and prevent tooth decay *in vivo* via intrinsic catalytic activity. Nat Commun 9:2920.

5. Bolyen E, Rideout JR, Dillon MR, Bokulich NA, Abnet CC, Al-Ghalith GA, Alexander H, Alm EJ, Arumugam M, Asnicar F, Bai Y, Bisanz JE, Bittinger K, Brejnrod A, Brislawn CJ, Brown CT, Callahan BJ, Caraballo-Rodríguez AM, Chase J, Cope EK, Da Silva R, Diener C, Dorrestein PC, Douglas GM, Durall DM, Duvallet C, Edwardson CF, Ernst M, Estaki M, Fouquier J, Gauglitz JM, Gibbons SM, Gibson DL, Gonzalez A, Gorlick K, Guo J, Hillmann B, Holmes S, Holste H, Huttenhower C, Huttley GA, Janssen S, Jarmusch AK, Jiang L, Kaehler BD, Kang KB, Keefe CR, Keim P, Kelley ST, Knights D, Koester I, Kosciolek T, Kreps J, Langille MGI, Lee J, Ley R, Liu Y-X, Loftfield E, Lozupone C, Maher M, Marotz C, Martin BD, McDonald D, McIver LJ, Melnik AV, Metcalf JL, Morgan SC, Morton JT, Naimey AT, Navas-Molina JA, Nothias LF, Orchanian SB, Pearson T, Peoples SL, Petras D, Preuss ML, Pruesse E, Rasmussen LB, Rivers A, Robeson MS, Rosenthal P, Segata N, Shaffer M, Shiffer A, Sinha R, Song SJ, Spear JR, Swafford AD, Thompson LR, Torres PJ, Trinh P, Tripathi A, Turnbaugh PJ, Ul-Hasan S, van der Hooft JJJ, Vargas F, Vázquez-Baeza Y, Vogtmann E, von Hippel M, Walters W, Wan Y, Wang M, Warren J, Weber KC, Williamson CHD, Willis AD, Xu ZZ, Zaneveld JR, Zhang Y, Zhu Q, Knight R, Caporaso JG. 2019. Reproducible, interactive, scalable and extensible microbiome data science using QIIME 2. Nat Biotechnol 37:852–857.

6. Benítez-Páez A, Belda-Ferre P, Simón-Soro A, Mira A. 2014. Microbiota diversity and gene expression dynamics in human oral biofilms. BMC Genomics 15:311.

7. Callahan BJ, McMurdie PJ, Rosen MJ, Han AW, Johnson AJA, Holmes SP. 2016. DADA2: High-resolution sample inference from Illumina amplicon data. Nat Methods 13:581–583.

8. Hoogenkamp MA, Crielaard W, Krom BP. 2015. Uses and limitations of green fluorescent protein as a viability marker in *Enterococcus faecalis*: An observational investigation. J Microbiol Methods 115:57–63.

9. Hartmann R, Jeckel H, Jelli E, Singh PK, Vaidya S, Bayer M, Rode DKH, Vidakovic L, Díaz-Pascual F, Fong JCN, Dragoš A, Lamprecht O, Thöming JG, Netter N, Häussler S, Nadell CD, Sourjik V, Kovács ÁT, Yildiz FH, Drescher K. 2021. Quantitative image analysis of microbial communities with BiofilmQ. Nat Microbiol 6:151–156.
